# Design and Synthesis of Novel Dehydroepiandrosterone Analogues as Potent Antiproliferative Agents

**DOI:** 10.3390/molecules23092243

**Published:** 2018-09-03

**Authors:** Xing Huang, Qing-Kun Shen, Hong-Jian Zhang, Jia-Li Li, Yu-Shun Tian, Zhe-Shan Quan

**Affiliations:** Key Laboratory of Natural Resources and Functional Molecules of the Changbai Mountain, Affiliated Ministry of Education, College of Pharmacy, Yanbian University, Yanji 133002, China; 2016010676@ybu.edu.cn (X.H.); 2016001065@ybu.edu.cn (Q.-K.S.); zhjzhishixiang@163.com (H.-J.Z.); 2016010667@ybu.edu.cn (J.-L.L.)

**Keywords:** dehydroepiandrosterone, 1,2,3-Triazoles, antiproliferative, synthesis

## Abstract

The aim of the present study was to determine the cytotoxic effects of a series of novel dehydroepiandrosterone derivatives containing triazole at the C_16_ position on human cancer cells. The cancer cells used in the present study were A549, Hela, HepG-2, BEL7402, MCF-7, and HCT116. Several of the synthesised compounds exhibited potent antiproliferative effects. The most promising compound was (*E*)-3-hydroxy-16-((1-(4-iodophenyl)-1*H*-1,2,3-triazole-4-yl)methylene)-10,13-dimet-hyl-1,3,4,7,8,9,10,11,12,13,15,16-dodecahydro-2*H*-cyclopenta[a]phenanthren-17(14)-one (compound **2n**), which showed considerably high antiproliferative activity in the HepG-2 cell line, with an IC_50_ value of 9.10 µM, and considerably high activity against the MCF-7 cell line, with an IC_50_ value of 9.18 µM. Flow cytometry assays demonstrated that compound **2n** exerted antiproliferative effects by arresting cells in the G2 phase of the cell cycle and inducing apoptosis.

## 1. Introduction

Cancer is one of the leading causes of death worldwide [1]. Because of its severity, cancer is considered one of the greatest social and economic concerns for the public health-care system [2]. Over the years, several anticancer drugs have been developed with excellent cytotoxicity such as paclitaxel and cisplatin [2].

Many anticancer drugs are nonselective, with paclitaxel and cisplatin being no exception. Therefore, their use is limited because of their inability to differentiate between normal cells and cancerous cells. Despite several advances made towards the diagnosis, prevention, and cure of cancer, it remains one of the major causes of human morbidity and mortality worldwide. Therefore, there is a requirement for safer and more effective anticancer drugs, which is a challenge for medicinal chemists [3].

More than 60% of all clinically used anticancer drugs originate from natural sources [4]. To develop selective drugs, many natural products are modified. Steroids are important physiological and pharmacological regulators of cell growth and survival. There are several examples in the literature that indicate the use of natural and synthetic steroids for the treatment of sex-hormone-dependent cancer tumours, including breast cancer [5], endometrial cancer [6], and cervical cancer [7]. These hormone-dependent tumour cells can control tumour growth through hormone receptor antagonists [5,6,7]. Different types of steroids have been modified as cytotoxic and cytostatic (antiproliferative) anticancer agents. The majority of steroidal anticancer drugs have been developed as enzyme inhibitors and cytotoxic drugs.

Recent years have seen an extensive focus on the modification of steroids. The rational modifications of the perhydrocyclopentanophenanthrene nucleus of steroids have yielded several important antiproliferative lead molecules. Exemestane, 2-methoxyestradiol, abiraterone, and fulvestrant are some of the successful leads that have emerged from steroidal pharmacophores [2].

**Figure 1:**
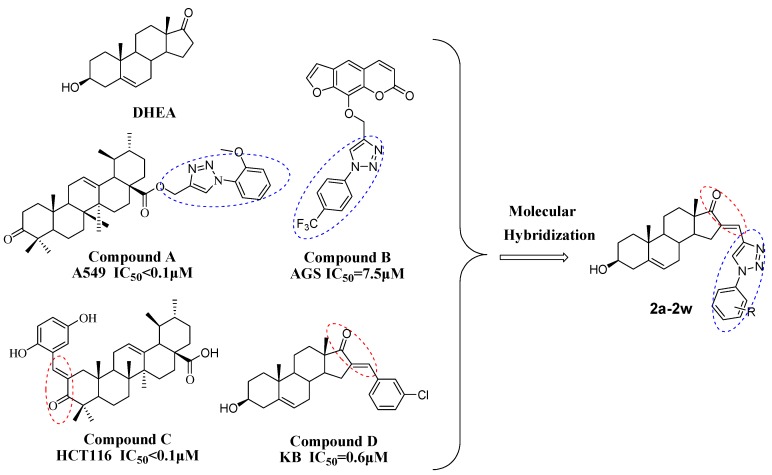
Design of target compound **2a**–**2w**.

In order to maintain preselected characteristics of the lead compounds, the molecular hybridization was a preferred choice for medicinal chemists [8,9,10]. Dehydroepiandrosterone (DHEA) (Figure 1) is secreted by the adrenal glands [11]. Other DHEA derivatives have also been reported as having antiproliferative properties in ovarian cancer cells (ES-2), human lung cells (A549), and liver carcinoma cell (HepG-2) lines [12]. Heterocyclic compounds containing 1,2,3-triazoles display a broad spectrum of biological activities, including antiproliferative [13,14,15]. Additionally, 1,2,3-triazole fragment is widely applied in organic, medicinal, and material sciences [14].

The 2-(substituted benzylidene) derivatives of C-3 oxidized ursolic acid (compound C) and 16-(substituted benzylidene) derivatives of DHEA (compound D) can be utilised as potent anticancer agents [16,17]. Chemically, compound C and compound D are open-chain precursors of flavonoids and isoflavonoids in which two aromatic rings are linked by a three-carbon α-β unsaturated carbonyl system [16]. The cytotoxic potential of the described compounds is mainly attributed to the position and nature of the substituted group on the benzylidene pendant [17].

Therefore, α-β unsaturated androsterone derivatives containing the exocyclic double bond at the C_16_ position could be utilised as potentially potent chemotherapeutic agents. The 1,2,3-triazoles can also increase the antitumour activity of the compound. In the present study, we utilised the molecular hybridization principle and designed, synthesised, and evaluated the antitumour biological activity of a series of new compounds that contained a 1,2,3-triazole between the α,β-unsaturated carbonyl group and phenyl, and then evaluated any antitumour biological activities.

## 2. Results and Discussion

### 2.1. Chemistry

Compounds **1a**–**1w** were obtained in three synthetic steps, with overall yields from 40% to 61%, based on procedures described in references [18,19,20,21]. In the first step, the substituted aniline diazotisation generated substituted 1-azidobenzene, and in the second step the substituted 1-azidobenzene with propargyl alcohol and CuSO_4_·5H_2_O and L-ascorbic acid sodium salt generated substituted phenyltriazole. The last step involved phenyltriazole oxidation by MnO_2_. From these three steps, compounds **1a**–**1w** were obtained. Compounds **2a**–**2w** were obtained by treating DHEA with different compounds **1a**–**1w** under the catalysis of KOH in anhydrous C_2_H_5_OH, with 90–98% yield.

### 2.2. In Vitro Antiproliferative Activity

All synthesised compounds were evaluated for their antiproliferative activities in vitro against six human cancer cell lines (A549, Hela, HepG-2, BEL7402, MCF-7 and HCT116) and compared with those of DHEA and 5-fluorouracil (5-FU). Cells were allowed to proliferate in the presence of the tested compounds for 48 h and the results are presented as the inhibition rate (Table 1) or IC_50_ values (Table 2). Some of the synthesised compounds showed highly significant antiproliferative effects. Among the compounds tested, compound **2n** exhibited better bioactivities against HepG-2 in vitro than 5-FU. Compound **2n** exhibited potent activity against HepG-2, HCT116, and MCF-7, with IC_50_ values of 9.10, 31.04, and 9.18 µM, respectively. Compound **2h** exhibited potent activity against A549, Hela, BEL7402, HCT116, and MCF-7, with IC_50_ values of 17.46, 15.11, 15.96, 11.86, and 14.93 µM, respectively.

### 2.3. Structure Activity Relationship Studies

Based on an overall comparison, the compounds derived from structures with electron-withdrawing substituents on the 1,2,3-triazole ring exhibited potent activity, whereas those with electron-donating substituents on the 1,2,3-triazole ring displayed no apparent activity against the six cancer cell lines. For the 3-substituent compounds, only 3-F substitution exhibited potent activity, and all 2-substituent compounds showed no significant activity. The special 3,4-Cl_2_ replacement exhibited potent activity.

Compound **2n** displayed the highest activity against HepG-2 and MCF-7; however, in other cancer cells, this compound exhibited lower activity and was not toxic in human normal liver (L02) cells with good selectivity. Compound **2k** displayed activity only against HepG-2, and for other cancer cells, this compound exhibited weaker activity. Compound **2h** displayed activity against all cancer cells except for HepG-2.

### 2.4. Selective Inhibition of Cancer Cell Growth by Compounds ***2c***, ***2d*** and ***2n***

Lack of selective cytotoxicity is the main factor that restricts the dose of most conventional chemotherapeutic agents [22]. We compared the toxicities of compounds **2c**, **2d,** and **2n** with 5-FU in L02 cells. Selectivity indexes between cancer cells and L02 cells were calculated. As shown in Table 3, compound **2n** exhibited more than a 10.99-fold and more than a 10.89-fold higher selectivity for HepG-2 and MCF-7 cells, respectively, than that for L02 cells; this selectivity displayed by compound **2n** was significantly higher than that displayed by 5-FU.

### 2.5. Compound ***2n*** Induces HepG-2 Cell Cycle Arrest

**Figure 2:**
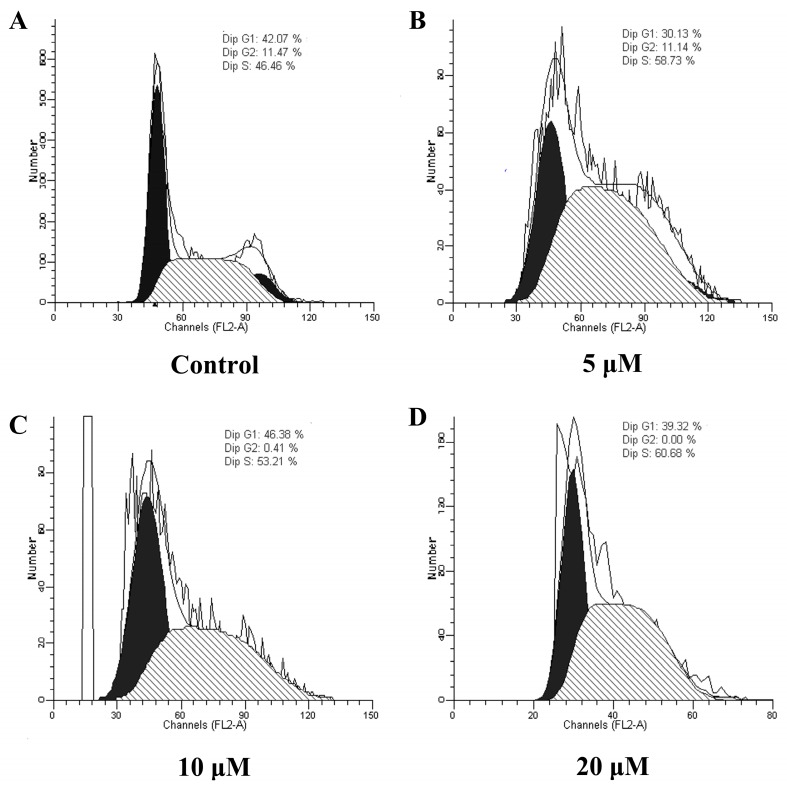
Flow cytometry analyses of cell cycle distribution of HepG-2 cancer cell after treatment of compound **2n** (5.0 Μm (**B**), 10.0 μM (**C**), 20.0 μM (**D**), and no treatment (Ctrl) (**A**) as a reference control for 48 h).

Numerous cytotoxic compounds exert their antiproliferative effect by inducing cell cycle arrest (at a particular cell cycle checkpoint), apoptosis, or both [23]. These mechanisms are considered to be effective anticancer strategies [24]. In the present study, flow cytometry was used to determine whether compound **2n** mediated the inhibition of growth and whether proliferation was associated with apoptosis. HepG-2 cell lines were treated with compound **2n** at concentrations of 5.0, 10.0, and 20.0 µM for 48 h, and the results are shown in Figure 2. The proportion of cells in the G2 phase decreased from 11.47% (control) to 11.14% (5.0 µM), 0.41% (10.0 µM), and 0.0% (20.0 µM). The rate of change in G2 phases showed a significant difference with the increasing concentration of compound **2n**. This finding suggests that compound **2n** significantly reduces the proportion of cells in the mitotic G2 phase.

### 2.6. Compound ***2n*** Induces HepG-2 Cell Apoptosis

**Figure 3:**
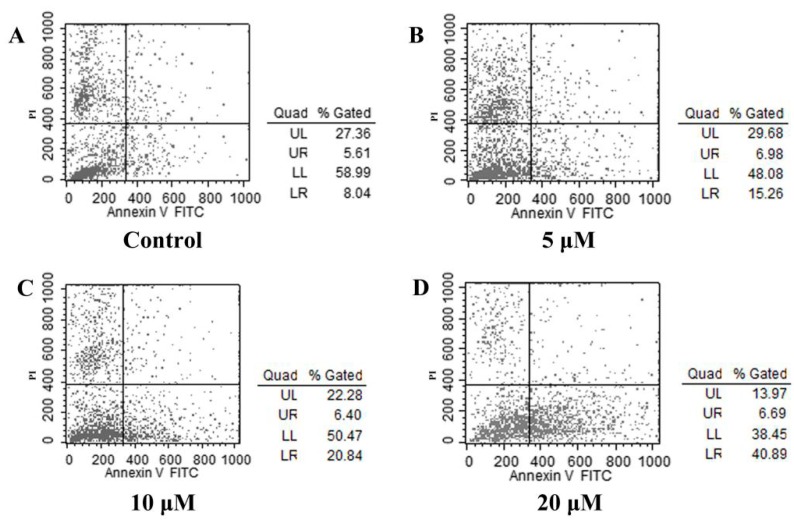
Apoptosis induction in HepG-2 cancer cell after treatment of compound **2n** (5.0 μM (**B**), 10.0 μM (**C**), 20.0 Μm (**D**), and no treatment (Ctrl) (**A**) as a reference control for 48 h).

Cell apoptosis analysis was performed to determine whether compound **2n** induced apoptosis of cells. As shown in Figure 3, the percentage of total apoptotic cells (right quadrants) increased from 13.65% (control) to 22.24% (5.0 µM), 27.24% (10.0 µM), and 47.86% (20.0 µM). This suggests that compound **2n** induced apoptosis and caused a marked increase in apoptosis in a concentration-dependent manner.

## 3. Experimental Section

### 3.1. General Information

Petroleum ether (PE), ethyl acetate (EA), ethanol (EtOH), and other reagents were obtained commercially and were used without further purification. Solvents were dried according to standard procedures. Reactions were monitored by thin-layer chromatography (TLC) on silica gel plates. ^1^H-NMR and ^13^C-NMR spectra were measured on an AV-300 (Bruker, Flawil, Switzerland), and all chemical shifts were given in ppm relative to TMS. Mass spectra were measured on an HP1100LC (Agilent Technologies, Palo Alto, CA, USA). High resolution mass spectra were measured on a MALDI-TOF/TOF mass spectrometer (Bruker Daltonik, Bremen, Germany).

### 3.2. Chemistry

#### 3.2.1. General Procedure for Preparation of **2a**–**2w**

**Scheme 1:**
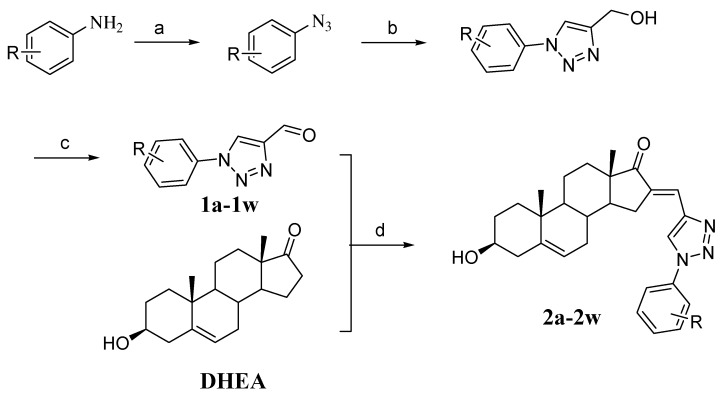
Reagents and conditions: (**a**) (i) NaNO_2_, HCl, 0 °C, 30 min; (ii) NaN_3_, H_2_O, 0 °C, 2–4 h; (**b**) propargyl alcohol, CuSO_4_, sodium ascorbate, H_2_O: *t*-butanol=1:1, 24 h, rt; (**c**) MnO_2_/EtOAc, 1 h, rt; (**d**) KOH/C_2_H_5_OH, overnight, reflux.

A mixture of dehydroepiandrosterone (DHEA) (2.0 mmol), aromatic aldehydes (2.1 mmol), and KOH (2.0 mmol) in EtOH (20 mL) was heated under reflux for about 1 h. After completion of the reaction as evident from TLC, the reaction solution was condensed under reduced pressure and the solid residue was transferred to 15 mL of water with 15 mL EA. The EA layer was washed with water (5 mL × 3) and saline (5 mL × 3) and dried using anhydrous MgSO_4_. The mixture was then purified by normal-phase column chromatography (PE:EA = 5:2) to obtain the target compounds **2a**–**2w** (Scheme 1).

*(E)-3-hydroxy-10,13-dimethyl-16-((1-phenyl-1H-1,2,3-triazol-4-yl)methylene)-1,3,4,7,8,9,10,11,12,13,15,16-dodecahydro-2H-cyclopenta[a]phenanthren-17(14H)-one* (**2a**) Yield 90%. ^1^H-NMR (300 MHz, CDCl_3_) δ 8.11 (s, 1H, triazole-H), 7.80 (s, 1H,-CO-C=CH), 7.77 (s, 1H, Ar-H), 7.58 (t, *J* = 6 Hz, 2H, Ar-H), 7.50 (t, *J* = 6 Hz, 2H, Ar-H), 5.44 (s, 1H, C_6_-H), 3.57 (s, 1H, -OH), 3.22 (dd, *J*_1_ = 15 MHz, *J*_2_ = 6 Hz, 1H, C_3_-H), 2.57–2.47 (m, 4H), 2.06–1.41 (m, 12H), 1.18–1.10 (m, 4H), 1.01 (m, 3H); ^13^C-NMR (75 MHz, CDCl_3_) δ 213.80, 149.37, 146.34, 142.43, 141.38, 134.59, 133.81, 127.47, 125.33, 125.04, 124.98, 75.49, 55.05, 54.07, 52.36, 47.05, 41.95, 41.47, 36.27, 35.91, 35.67, 34.29, 25.09, 34.23, 19.05; ESI-HR MS (*m/z*): calcd. for C_28_H_34_N_3_O_2_^+^ [M + H]^+^: 444.2646; found: 444.2651. Purity: 98.066% by HPLC (A: H_2_O; B: methanol, graded: 60–100%), t_R_ 15.873 min, λ: 292 nm.

*(E)-16-((1-(2-fluorophenyl)-1H-1,2,3-triazol-4-yl)methylene)-3-hydroxy-10,13-dimethyl-1,3,4,7,8,9,10,11,12,13,15,16-dodecahydro-2H-cyclopenta[a]phenan-thren-17(14H)-one* (**2b**) Yield 98%. ^1^H-NMR (300 MHz, CDCl_3_) δ 8.71 (s, 1H, triazole-H), 8.08 (s, 1H,-CO-C=CH), 7.86 (t, *J* = 6 Hz, 2H, Ar-H), 7.61–7.39 (m, 3H, Ar-H), 7.50 (t, *J* = 6 Hz, 2H, Ar-H), 5.53 (s, 1H, C_6_-H), 4,53 (s, 1H, -OH), 3.07 (dd, *J*_1_ = 15 Hz, *J*_2_ = 6 Hz, 1H, C_3_-H), 2.42–2.17 (m, 4H), 1.90–1.36 (m, 12H), 1.04 (s, 4H), 0.94 (m, 3H); ^13^C-NMR (75 MHz, DMSO-*d*_6_) δ 208.50, 143.89, 141.87, 137.99, 128.10, 128.05, 126.79, 126.05, 126.01, 120.47, 120.03, 117.74, 117.49, 70.43, 50.25, 48.97, 47.54, 42.69, 37.31, 36.77, 31.88, 31.63, 31.13, 30.78, 29.53, 20.47, 19.66, 14.43; ESI-HR MS (*m/z*): calcd. for C_28_H_33_FN_3_O_2_^+^ [M + H]^+^: 462.2551; found: 462.2558. Purity: 100% by HPLC (A: H_2_O; B: methanol, graded: 80–100%), t_R_ 5.820 min, λ: 292 nm.

*(E)-16-((1-(3-fluorophenyl)-1H-1,2,3-triazol-4-yl)methylene)-3-hydroxy-10,13-dimethyl-1,3,4,7,8,9,10,11,12,13,15,16-dodecahydro-2H-cyclopenta[a]phenanthren-17(14H)-one* (**2c**) Yield 98%. ^1^H-NMR (300 MHz, DMSO-d_6_) δ 8.95 (s, 1H, triazole-H), 7.86 (t, *J* = 6 Hz, 1H, -CO-C=CH), 7.69–7.56 (m, 2H, Ar-H), 7.47 (t, *J* = 6 Hz, 2H, Ar-H), 7.34 (s, 1H, Ar-H), 5.33 (s, 1H, C_6_-H), 4.61 (d, *J* = 3 Hz, 1H, -OH), 4.08 (dd, *J*_1_ = 9 Hz, *J*_2_ = 6 Hz, 1H, C_3_-H), 3.17 (d, *J* = 3 Hz, 2H), 3.07–2.99 (m, 1H), 2.20–2.12 (m, 3H), 1.83–1.66 (m, 6H), 1.53–1.30 (m, 4H), 1.01 (s, 4H), 0.89 (s, 3H); ^13^C-NMR (75 MHz, DMSO-*d*_6_) δ 213.37, 169.30, 149.37, 146.45, 142.61, 136.36, 128.27, 124.97, 120.86, 120.58, 120.27, 112.99, 112.64, 75.29, 55.03, 53.97, 52.30, 47.22, 41.98, 41.50, 36.40, 36.31, 35.95, 35.72, 34.26, 25.11, 24.30, 19.09; ESI-HR MS (*m/z*): calcd. for C_28_H_33_FN_3_O_2_^+^ [M + H]^+^: 462.2551; found: 462.2560. Purity: 100% by HPLC (A: H_2_O; B: methanol, graded: 60–100%), t_R_ 17.187 min, λ: 292 nm.

*(E)-16-((1-(4-fluorophenyl)-1H-1,2,3-triazol-4-yl)methylene)-3-hydroxy-10,13-dimethyl-1,3,4,7,8,9,10,11,12,13,15,16-dodecahydro-2H-cyclopenta[a]phenanthren-17(14H)-one* (**2d**) Yield 96%. ^1^H-NMR (300 MHz, CDCl_3_) δ 8.06 (s, 1H, triazole-H), 7.78–7.74 (m, 2H, Ar-H), 7.47 (s, 1H, Ar-H), 7.29 (d, *J* = 6 Hz, 2H, Ar-H), 5.44 (s, 1H, C_6_-H), 3.57 (s, 1H, -OH), 3.22 (dd, *J*_1_ = 18 Hz, *J*_2_ = 6 Hz, 1H, C_3_-H), 2.57–2.46 (m, 1H), 2.40–2.29 (m, 3H), 2.06–1.40 (m, 12H), 1.30–1.15 (m, 1H), 1.10 (m, 4H), 1.01 (m, 3H); ^13^C-NMR (75 MHz, CDCl_3_) δ 209.32, 144.98, 141.09, 138.21, 122.69, 122.66, 122.57, 120.93, 119.56, 117.05, 116.74, 71.59, 50.37, 49.41, 47.71, 42.24, 37.17, 26.73, 31.59, 31.51, 31.21, 30.98, 29.70, 20.43, 19.47, 14.29; ESI-HR MS (*m/z*): calcd. for C_28_H_33_FN_3_O_2_^+^ [M + H]^+^: 462.2551; found: 462.2547. Purity: 97.271% by HPLC (A: H_2_O; B: methanol, graded: 80–100%), t_R_ 5.987 min, λ: 292 nm.

*(E)-16-((1-(2-chlorophenyl)-1H-1,2,3-triazol-4-yl)methylene)-3-hydroxy-10,13-di-methyl-1,3,4,7,8,9,10,11,12,13,15,16-dodecahydro-2H-cyclopenta[a]phenan-thren-17(14H)-one* (**2e**) Yield 92%. ^1^H-NMR (300 MHz, CDCl_3_) δ 8.14 (s, 1H, triazole-H), 7.68–1,62 (m, 2H, Ar-H), 7.54–7.47(m, 3H, Ar-H), 5.43 (s, 1H, C_6_-H), 3.55 (s, 1H, -OH), 3.22 (dd, *J*_1_ = 18 Hz, *J*_2_ = 6 Hz, 1H, C_3_-H), 2.57–2.46 (m, 1H), 2.39–2.24 (m, 3H), 2.06–1.99 (m, 1H), 1.92–1.74 (m, 4H), 1.64–1.56 (m, 5H), 1.51–1.41 (m, 3H), 1.14–1.10 (m, 3H), 1.01 (s, 3H); ^13^C-NMR (75 MHz, CDCl_3_) δ 209.31, 144.08, 141.06, 138.04, 131.03, 130.88, 128.63, 128.06, 127.68, 126.55, 120.95, 119.75, 71.59, 50.38, 49.43, 47.72, 42.23, 37.17, 36.73, 31.58, 31.22, 30.97, 29.68, 20.43, 19.46, 14.29; ESI-HR MS (*m/z*): calcd. for C_28_H_33_ClN_3_O_2_^+^ [M + H]^+^: 478.2256; found: 478.2663. Purity: 98.081% by HPLC (A: H_2_O; B: methanol, graded: 80–100%), t_R_ 4.933 min, λ: 292 nm.

*(E)-16-((1-(3-chlorophenyl)-1H-1,2,3-triazol-4-yl)methylene)-3-hydroxy-10,13-dimethyl-1,3,4,7,8,9,10,11,12,13,15,16-dodecahydro-2H-cyclopenta[a]phenanthren-17(14H)-one* (**2f**) Yield 90%. ^1^H-NMR (300 MHz, CDCl_3_) δ 8.07 (s, 1H, triazole-H), 7.80 (s, 1H,-CO-C=CH), 7.68 (d, *J* = 3 Hz, 1H, Ar-H), 7.50–7.45 (m, 3H, Ar-H), 5.42 (s, 1H, C_6_-H), 3.55 (s, 1H, -OH), 3.20 (dd, *J*_1_ = 12 Hz, *J*_2_ = 3 Hz, 1H, C_3_-H), 2.53–2.47 (m, 1H), 2.37–2.25 (m, 3H), 2.04–1.98 (m, 2H), 1.90–1.82 (m, 4H), 1.75–1.70 (m, 2H), 1.52–1.44 (m, 4H), 1.15–1.08 (m, 4H), 0.99 (m, 3H); ^13^C-NMR (75 MHz, CDCl_3_) δ 209.37, 145.06, 141.04, 137.45, 135.70, 130.98, 129.15, 122.39, 120.90, 120.78, 119.53, 119.50, 118.98, 71.56, 50.34, 49.38, 47.73, 42.20, 37.15, 36.70, 31.53, 31.20, 31.17, 30.97, 29.70, 20.42, 19.46, 14.29; ESI-HR MS (*m/z*): calcd. for C_28_H_33_ClN_3_O_2_^+^ [M + H]^+^: 478.2256; found: 478.2680. Purity: 98.406% by HPLC (A: H_2_O; B: methanol, graded: 60–100%), t_R_ 19.453 min, λ: 292 nm.

*(E)-16-((1-(4-chlorophenyl)-1H-1,2,3-triazol-4-yl)methylene)-3-hydroxy-10,13-dimethyl-1,3,4,7,8,9,10,11,12,13,15,16-dodecahydro-2H-cyclopenta[a]phenanthren-17(14H)-one* (**2g**) Yield 97%. ^1^H-NMR (300 MHz, CDCl_3_) δ 8.09 (s, 1H, triazole-H), 7.75 (s, 1H,-CO-C=CH), 7.72 (s, 1H, Ar-H), 7.55 (d, *J* = 6 Hz, 2H, Ar-H), 7.46 (s, 1H, Ar-H), 5.43 (s, 1H, C_6_-H), 3.21 (dd, *J*_1_ = 15 Hz, *J*_2_ = 6 Hz, 1H, C_3_-H), 2.56–2.46 (m, 1H), 2.35–2.24 (m, 3H), 2.02–1.40 (m, 12H), 1.09 (m, 5H), 1.00 (s, 3H); ^13^C-NMR (75 MHz, CDCl_3_) δ 213.39, 149.39, 146.44, 142.52, 140.09, 138.92, 134.66, 128.13, 126.65, 124.96, 75.31, 55.04, 53.99, 52.30, 47.20, 41.98, 41.50, 36.32, 35.94, 35.70, 34.25, 25.12, 24.29, 23.56, 19.09; ESI-HR MS (*m/z*): calcd. for C_28_H_33_ClN_3_O_2_^+^ [M + H]^+^: 478.2256; found: 478.2563. Purity: 100% by HPLC (A: H_2_O; B: methanol, graded: 95–100%), t_R_ 2.860 min, λ: 292 nm.

*(E)-16-((1-(3,4-dichlorophenyl)-1H-1,2,3-triazol-4-yl)methylene)-3-hydroxy-10,13-dimethyl-1,3,4,7,8,9,10,11,12,13,15,16-dodecahydro-2H-cyclopenta[a]phenan-thren-17(14H)-one* (**2h**) Yield 98%. ^1^H-NMR (300 MHz, CDCl_3_) δ 8.08 (s, 1H, triazole-H), 7.94 (s, 1H,-CO-C=CH), 7.66 (s, 2H, Ar-H), 7.45 (s, 1H, Ar-H), 5.44 (s, 1H, C_6_-H), 3.57 (s, 1H, -OH), 3.21 (dd, *J*_1_ = 15 Hz, *J*_2_ = 6 Hz, 1H, C_3_-H), 2.57–2.46 (m, 1H), 2.38–2.25 (m, 3H), 2.06–2.02 (m, 1H), 1.92–1.69 (m, 5H), 1.64–1.55 (m, 4H), 1.50–1.40 (m, 2H), 1.18–1.10 (m, 4H), 1.00 (m, 3H); ^13^C-NMR (75 MHz, CDCl_3_) δ 209.19, 145.29, 141.08, 138.72, 135.65, 134.17, 133.30, 131.62, 122.29, 122.13, 120.91, 119.52, 119.17, 71.61, 50.38, 49.38, 47.72, 42.23, 37.17, 36.73, 31.59, 31.51, 31.22, 30.99, 29.71, 20.43, 19.46, 14.28; ESI-HR MS (*m/z*): calcd. for C_28_H_32_Cl_2_N_3_O_2_^+^ [M + H]^+^: 512.1866; found: 512.1859. Purity: 98.857% by HPLC (A: H_2_O; B: methanol, graded: 60–100%), t_R_ 22.315 min, λ: 292 nm.

*(E)-16-((1-(2-bromophenyl)-1H-1,2,3-triazol-4-yl)methylene)-3-hydroxy-10,13-dimethyl-1,3,4,7,8,9,10,11,12,13,15,16-dodecahydro-2H-cyclopenta[a]phenan-thren-17(14H)-one* (**2i**) Yield 98%. ^1^H-NMR (300 MHz, CDCl_3_) δ 8.27 (s, 1H, triazole-H), 7.80 (d, *J* = 6 Hz, 1H, -CO-C=CH), 7.61–7.45 (m, 4H, Ar-H), 5.43 (s, 1H, C_6_-H), 3.22 (dd, *J*_1_ = 15 Hz, *J*_2_ = 6 Hz, 1H, C_3_-H), 3.25–3.18 (m, 1H), 2.55–2,46 (m, 1H), 2.35–2.24 (m, 3H), 1.93–1.88 (m, 3H), 1.82–1.58 (m, 6H), 1.51–1.45 (m, 3H), 1.09 (m, 4H), 1.01 (m, 3H); ^13^C-NMR (75 MHz, CDCl_3_) δ 209.34, 143.94, 141.14, 137.96, 136.11, 134.01, 131.48, 128.66, 128.10, 126.71, 120.85, 119.87, 118.57, 71.47, 50.34, 49.38, 47.71, 42.19, 37.17, 36.72, 31.53, 31.18, 30.95, 29.66, 20.42, 19.48, 14.30; ESI-HR MS (*m/z*): calcd. for C_28_H_33_BrN_3_O_2_^+^ [M + H]^+^: 522.1751; found: 522.1743. Purity: 99.701% by HPLC (A: H_2_O; B: methanol, graded: 60–100%), t_R_ 15.587 min, λ: 292 nm.

*(E)-16-((1-(3-bromophenyl)-1H-1,2,3-triazol-4-yl)methylene)-3-hydroxy-10,13-dimethyl-1,3,4,7,8,9,10,11,12,13,15,16-dodecahydro-2H-cyclopenta[a]phenan- thren-17(14H)-one* (**2j**) Yield 98%. ^1^H-NMR (300 MHz, CDCl_3_) δ 8.09 (s, 1H, triazole-H), 7.97 (d, *J* = 6 Hz, 1H, -CO-C=CH), 7.75 (dd, *J*_1_ = 6 Hz, *J*_2_ = 3 Hz, 1H, Ar-H), 7.63 (t, *J* = 3 Hz, 1H, Ar-H), 7.48–7.43 (m, 2H, Ar-H), 5.43 (s, 1H, C_6_-H), 3.57 (s, 1H, -OH), 3.22 (dd, *J*_1_ = 18 Hz, *J*_2_ = 6 Hz, 1H, C_3_-H), 2.57–2.46 (m, 1H), 2.38–2.25 (m, 3H), 2.06–1.86 (m, 9H), 1.52–1.40 (m, 3H), 1.15–1.06 (m, 4H), 1.01 (m, 3H); ^13^C-NMR (75 MHz, CDCl_3_) δ 209.31, 145.07, 141.06, 138.42, 137.55, 132.10, 131.22, 123.61, 123.42, 122.35, 120.91, 119.48, 119.10, 71.58, 50.35, 49.37, 47.72, 42.21, 37.16, 36.72, 31.56, 31.20, 30.98, 29.69, 20.42, 19.47, 14.29; ESI-HR MS (*m/z*): calcd. for C_28_H_33_BrN_3_O_2_^+^ [M + H]^+^: 522.1751; found: 522.1740. Purity: 98.860% by HPLC (A: H_2_O; B: methanol, graded: 60–100%), t_R_ 20.073 min, λ: 292 nm.

*(E)-16-((1-(4-bromophenyl)-1H-1,2,3-triazol-4-yl)methylene)-3-hydroxy-10,13-di-methyl-1,3,4,7,8,9,10,11,12,13,15,16-dodecahydro-2H-cyclopenta[a]phenan- thren-17(14H)-one* (**2k**) Yield 95%. ^1^H-NMR (300 MHz, CDCl_3_) δ 8.09 (s, 1H, triazole-H), 7.72–7.65 (m, 4H, Ar-H), 7.46–7.44 (dd, *J*_1_ = 6 Hz, *J*_2_ = 3 Hz, 1H, Ar-H), 5.44 (s, 1H, C_6_-H), 3.60–3.52 (m, 1H), 3.25–3.17 (m, 1H, C_3_-H), 2.56–2.45 (m, 1H), 2.38–2.24 (m, 3H), 2.02–1.39 (m, 12H), 1.14–1.1.09 (m, 4H), 1.00 (s, 3H); ^13^C-NMR (75 MHz, CDCl_3_) δ 208.48, 144.40, 141.96, 138.06, 135.98, 135.26, 124.45, 122.79, 122.18, 120.43, 120.12, 70.43, 50.24, 49.06, 48.93, 47.54, 42.70, 37.28, 36.77, 31.89, 31.60, 31.16, 30.87, 29.48, 19.67, 14.42; ESI-HR MS (*m/z*): calcd. for C_28_H_33_BrN_3_O_2_^+^ [M + H]^+^: 522.1751; found: 522.1743. Purity: 99.001% by HPLC (A: H_2_O; B: methanol, graded: 60–100%), t_R_ 19.640 min, λ: 292 nm.

*(E)-3-hydroxy-16-((1-(2-iodophenyl)-1H-1,2,3-triazol-4-yl)methylene)-10,13-dim-ethyl-1,3,4,7,8,9,10,11,12,13,15,16-dodecahydro-2H-cyclopenta[a]phenanthren-17(14H)-one* (**2l**) Yield 91%. ^1^H-NMR (300 MHz, CDCl_3_) δ 8.06 (s, 1H, triazole-H), 8.03 (d, *J* = 3 MHz, 1H, -CO-C=CH), 7.59 (m, 3H, Ar-H), 7.32 (s, 1H, Ar-H), 5.43 (s, 1H, C_6_-H), 3.56 (s, 1H, -OH), 3.23 (dd, *J*_1_ = 15 Hz, *J*_2_ = 6 Hz, 1H, C_3_-H), 2.56–2.43 (m, 1H), 2.35–2.24 (m, 3H), 2.03–1.73 (m, 5H), 1.56–1.42 (m, 4H), 1.18–1.10 (m, 4H), 1.01 (m, 3H); ^13^C-NMR (75 MHz, CDCl_3_) δ 209.28, 144.05, 141.11, 140.35, 139.65, 137.98, 133.47, 131.92, 129.38, 127.36, 126.54, 124.42, 120.88, 119.82, 117.47, 71.51, 50.37, 49.81, 49.41, 48.82, 47.71, 42.21, 37.17, 36.72, 31.55, 31.51, 31.20, 30.96, 30.89, 29.69, 20.41, 19.45, 14.29; ESI-HR MS (*m/z*): calcd. for C_28_H_33_IN_3_O_2_^+^ [M + H]^+^: 570.1612; found: 570.1595. Purity: 98.823% by HPLC (A: H_2_O; B: methanol, graded: 60–100%), t_R_ 15.273 min, λ: 292 nm.

*(E)-3-hydroxy-16-((1-(3-iodophenyl)-1H-1,2,3-triazol-4-yl)methylene)-10,13-dim-ethyl-1,3,4,7,8,9,10,11,12,13,15,16-dodecahydro-2H-cyclopenta[a]phenanthren-17(14H)-one* (**2m**) Yield 96%. ^1^H-NMR (300 MHz, CDCl_3_) δ 8.14 (s, 1H, triazole-H), 8.08 (s, 1H,-CO-C=CH), 7.87–7.77 (m, 2H, Ar-H), 7.33 (s, 1H, Ar-H), 5.44 (s, 1H, C_6_-H), 3.58 (s, 1H, -OH), 3.21 (dd, *J*_1_ = 15 Hz, *J*_2_ = 6 Hz, 1H, C_3_-H), 2.56–2.47 (m, 1H), 2.34–2.25 (m, 3H), 2.08–1.74 (m, 7H), 1.54–1.27 (m, 5H), 1.10 (m, 4H), 1.01 (m, 3H); ^13^C-NMR (75 MHz, CDCl_3_) δ 209.30, 145.06, 141.05, 138.40, 138.05, 137.41, 131.28, 129.31, 122.32, 120.94, 119.82, 119.49, 71.54, 50.44, 49.32, 47.67, 42.20, 37.23, 36.74, 31.58, 31.51, 31.28, 31.17, 31.06, 29.73, 20.45, 19.53, 14.36; ESI-HR MS (*m/z*): calcd. for C_28_H_33_IN_3_O_2_^+^ [M + H]^+^: 570.1612; found: 570.1604. Purity: 98.003% by HPLC (A: H_2_O; B: methanol, graded: 60–100%), t_R_ 20.673 min, λ: 292 nm.

*(E)-3-hydroxy-16-((1-(4-iodophenyl)-1H-1,2,3-triazol-4-yl)methylene)-10,13-dimethyl-1,3,4,7,8,9,10,11,12,13,15,16-dodecahydro-2H-cyclopenta[a]phenanthren-17(14H)-one* (**2n**) Yield 92%. ^1^H-NMR (300 MHz, CDCl_3_) δ 8.08 (s, 1H, triazole-H), 7.91 (d, *J* = 6 Hz, 1H, Ar-H), 7.53 (m, 3H, Ar-H), 5.44 (s, 1H, C_6_-H), 3.56 (s, 1H, -OH), 3.21 (dd, *J*_1_ = 15 Hz, *J*_2_ = 6 Hz, 1H, C_3_-H), 2.59–2.46 (m, 1H), 2.39–2.25 (m, 3H), 2.12–1.99 (m, 2H), 1.92–1.71 (m, 6H), 1.62–1.46 (m, 5H), 1.10 (s, 4H), 1.00 (m, 3H); ^13^C-NMR (75 MHz, CDCl_3_) δ 209.32, 145.12, 141.05, 139.02, 138.39, 136.27, 122.18, 122.07, 120.96, 119.45, 94.04, 71.62, 50.37, 49.40, 47.72, 42.21, 37.17, 36.73, 31.57, 31.51, 31.21, 30.98, 29.71, 20.43, 19.41, 14.30; ESI-HR MS (*m/z*): calcd. for C_28_H_33_IN_3_O_2_^+^ [M + H]^+^: 570.1612; found: 570.1601. Purity: 100% by HPLC (A: H_2_O; B: methanol, graded: 60–100%), t_R_ 20.460 min, λ: 292 nm.

*(E)-3-hydroxy-10,13-dimethyl-16-((1-m-tolyl-1H-1,2,3-triazol-4-yl)methylene)-1,3,4,7,8,9,10,11,12,13,15,16-dodecahydro-2H-cyclopenta[a]phenanthren-17(14H)-one* (**2o**) Yield 90%. ^1^H-NMR (300 MHz, CDCl_3_) δ 8.08 (s, 1H, triazole-H), 7.60 (s, 1H,-CO-C=CH), 7.55 (d, *J* = 6 Hz, 1H, Ar-H), 7.50–1.42 (m, 2H, Ar-H), 7.31 (s, 1H, Ar-H), 5.43 (s, 1H, C_6_-H), 3.57 (s, 1H, -OH), 3.22 (dd, *J*_1_ = 18 Hz, *J*_2_ = 6 Hz, 1H, C_3_-H), 2.56–2.47 (m, 4H), 2.35–2.25 (m, 3H), 2.06–1.87 (m, 5H), 1.83–1.74 (m, 3H), 1.61–1.40 (m, 8H), 1.13–1.06 (m, 4H), 1.01 (m, 3H); ^13^C-NMR (75 MHz, CDCl_3_) δ 209.40, 144.72, 141.09, 140.16, 137.90, 136.59, 129.87, 129.65, 122.62, 121.24, 120.92, 119.89, 117.70, 71.56, 50.38, 49.42, 47.71, 42.23, 37.17, 36.72, 31.56, 31.53, 31.20, 30.98, 29.68, 21.43, 20.43, 19.46, 14.29; ESI-HR MS (*m/z*): calcd. for C_29_H_36_N_3_O_2_^+^ [M + H]^+^: 458.2802; found: 458.2804. Purity: 100% by HPLC (A: H_2_O; B: methanol, graded: 680–100%), t_R_ 6.500 min, λ: 292 nm.

*(E)-3-hydroxy-10,13-dimethyl-16-((1-p-tolyl-1H-1,2,3-triazol-4-yl)methylene)-1,3,4,7,8,9,10,11,12,13,15,16-dodecahydro-2H-cyclopenta[a]phenanthren-17(14H)-one* (**2p**) Yield 96%. ^1^H-NMR (300 MHz, CDCl_3_) δ 8.06 (s, 1H, triazole-H), 7.66 (s, 1H,-CO-C=CH), 7.63 (s, 1H, Ar-H), 7.48 (s, 1H, Ar-H), 7.35 (t, *J* = 6 Hz, 2H, Ar-H), 5.44 (s, 1H, C_6_-H), 3.57 (s, 1H, -OH), 3.22 (dd, *J*_1_ = 15 MHz, *J*_2_ = 6 Hz, 1H, C_3_-H), 2.56–2.46 (m, 4H), 2.41–2.25 (m, 3H), 2.06–1.44 (m, 12H), 1.14–1.10 (m, 4H), 1.00 (s, 3H); ^13^C-NMR (75 MHz, CDCl_3_) δ 209.44, 144.69, 141.05, 139.33, 137.85, 134.34, 130.37, 122.57, 120.96, 120.52, 119.91, 71.60, 50.35, 49.40, 47.71, 42.20, 31.17, 36.72, 31.50, 31.19, 30.97, 29.68, 21.15, 20.43, 19.47, 14.30; ESI-HR MS (*m/z*): calcd. for C_29_H_36_N_3_O_2_^+^ [M + H]^+^: 458.2802; found: 458.2811. Purity: 97.937% by HPLC (A: H_2_O; B: methanol, graded: 60–100%), t_R_ 18.167 min, λ: 292 nm.

*(E)-3-hydroxy-16-((1-(2-methoxyphenyl)-1H-1,2,3-triazol-4-yl)methylene)-10,13-dimethyl-1,3,4,7,8,9,10,11,12,13,15,16-dodecahydro-2H-cyclopenta[a]phenan-thren-17(14H)-one* (**2q**) Yield 96%. ^1^H-NMR (300 MHz, CDCl_3_) δ 8.27 (s, 1H, triazole-H), 7.82 (d, *J* = 6 Hz, 1H,-CO-C=CH), 7.47 (t, *J* = 9 Hz, 2H, Ar-H), 7.15 (t, *J* = 9 Hz, 2H, Ar-H), 5.44 (s, 1H, C_6_-H), 3.93 (s, 3H, -OCH_3_), 3.23 (dd, *J*_1_ = 18 Hz, *J*_2_ = 3 Hz, 1H, C_3_-H), 2.55–2.47 (m, 1H), 2.33–2.24 (m, 3H), 2.06–1.88 (m, 3H), 1.82–1.62 (m, 5H), 1.58–1.28 (m, 5H), 1.10–1.01 (m, 8H); ^13^C-NMR (75 MHz, DMSO-*d*_6_) δ 209.55, 151.06, 143.74, 141.08, 137.31, 130.39, 126.93, 125.90, 125.31, 121.35, 120.98, 120.28, 112.36, 71.57, 56.07, 50.38, 49.47, 47.70, 42.23, 37.17, 36.73, 31.57, 31.21, 30.96, 29.66, 20.44, 19.46, 14.31; ESI-HR MS (*m/z*): calcd. for C_29_H_36_N_3_O_3_^+^ [M + H]^+^: 474.2751; found: 474.2762. Purity: 98.888% by HPLC (A: H_2_O; B: methanol, graded: 60–100%), t_R_ 15.440 min, λ: 292 nm.

*(E)-3-hydroxy-16-((1-(3-methoxyphenyl)-1H-1,2,3-triazol-4-yl)methylene)-10,13-dimethyl-1,3,4,7,8,9,10,11,12,13,15,16-dodecahydro-2H-cyclopenta[a]phenan-thren-17(14H)-one* (**2r**) Yield 95%. ^1^H-NMR (300 MHz, CDCl_3_) δ 8.09 (s, 1H, triazole-H), 7.49 (s, 1H,-CO-C=CH), 7.45 (d, *J* = 3 Hz, 1H, Ar-H), 7.38 (d, *J* = 3 Hz, 1H, Ar-H), 7.32 (s, 1H, Ar-H), 7.02 (dd, *J*_1_ = 9 Hz, *J*_2_ = 3 Hz, 1H, Ar-H), 5.45 (s, 1H, C_6_-H), 3.92 (s, 3H, -OCH_3_), 3.57 (s, 1H, -OH), 3.21 (dd, *J*_1_ = 15 MHz, *J*_2_ = 6 MHz, 1H, C_3_-H), 2.57–2.46 (m, 1H), 2.36–2.25 (m, 3H), 2.07–1.40 (m, 13H), 1.18–1.10 (m, 3H), 1.01 (m, 3H); ^13^C-NMR (75 MHz, CDCl_3_) δ 209.35, 160.67, 144.79, 141.07, 138.03, 137.64, 130.67, 122.59, 120.94, 119.76, 114.76, 112.42, 106.56, 71.57, 55.67, 50.37, 49.48, 47.71, 42.23, 37.17, 36.72, 31.58, 31.51, 31.20, 30.97, 29.68, 20.43, 19.47, 14.30; ESI-HR MS (*m/z*): calcd. for C_29_H_36_N_3_O_3_^+^ [M + H]^+^: 474.2751; found: 474.2754. Purity: 100% by HPLC (A: H_2_O; B: methanol, graded: 60–100%), t_R_ 16.440 min, λ: 292 nm.

*(E)-3-hydroxy-16-((1-(4-methoxyphenyl)-1H-1,2,3-triazol-4-yl)methylene)-10,13-dimethyl-1,3,4,7,8,9,10,11,12,13,15,16-dodecahydro-2H-cyclopenta[a]phenan-thren-17(14H)-one* (**2s**) Yield 97%. ^1^H-NMR (300 MHz, CDCl_3_) δ 8.01 (s, 1H, triazole-H), 7.67 (s, 1H, -CO-C=CH), 7.64 (s, 1H, Ar-H), 7.47 (s, 1H, Ar-H), 7.05 (d, *J* = 6 Hz, 2H, Ar-H), 5.42 (s, 1H, C_6_-H), 3.88 (s, 3H, -OCH_3_), 3.55 (s, 1H, -OH), 3.19 (dd, *J*_1_ = 18 Hz, *J*_2_ = 6 Hz, 1H, C_3_-H), 2.54–2.23 (m, 4H), 2.05–1.44 (m, 12H), 1.08 (s, 4H), 0.99 (s, 3H); ^13^C-NMR (75 MHz, CDCl_3_) δ 213.78, 164.71, 149.14, 146.34, 142.15, 134.76, 127.85, 126.97, 125.16, 125.03, 119.59, 75.46, 60.43, 55.05, 54.07, 52.35, 47.07, 41.96, 41.47, 36.29, 35.90, 35.67, 34.26, 25.06, 24.24, 19.06; ESI-HR MS (*m/z*): calcd. for C_29_H_36_N_3_O_3_^+^ [M + H]^+^: 474.2751; found: 474.2759. Purity: 100% by HPLC (A: H_2_O; B: methanol, graded: 95–100%), t_R_ 2.527 min, λ: 292 nm.

*(E)-16-((1-(3,4-dimethoxyphenyl)-1H-1,2,3-triazol-4-yl)methylene)-3-hydroxy-10,13-dimethyl-1,3,4,7,8,9,10,11,12,13,15,16-dodecahydro-2H-cyclopenta[a]phenan-thren-17(14H)-one* (**2t**) Yield 93%. ^1^H-NMR (300 MHz, CDCl_3_) δ 8.04 (s, 1H, triazole-H), 7.47 (s, 1H,-CO-C=CH), 7.39 (d, *J* = 3 Hz, 1H, Ar-H), 7.20 (dd, *J*_1_ = 9 Hz, *J*_2_ = 3 Hz, 1H, Ar-H), 7.00 (d, *J* = 3 Hz, 1H, Ar-H), 5.44 (s, 1H, C_6_-H), 3.99 (s, 3H, -OCH_3_), 3.97(s, 3H, -OCH_3_), 3.21 (dd, *J*_1_ = 15 Hz, *J*_2_ = 6 Hz, 1H, C_3_-H), 2.56–2.25 (m, 5H), 2.05–1.98 (m, 2H), 1.91–1.74 (m, 5H), 1.62–1.44 (m, 4H), 1.17–1.00 (m, 8H); ^13^C-NMR (75 MHz, CDCl_3_) δ 209.49, 149.89, 144.68, 141.08, 137.88, 130.19, 122.76, 120.94, 119.85, 112.55, 111.25, 105.06, 71.59, 56.27, 50.36, 49.41, 47.71, 42.21, 37.16, 36.72, 31.56, 31.20, 30.97, 29.67, 20.42, 19.46, 14.30; ESI-HR MS (*m/z*): calcd. for C_30_H_38_N_3_O_4_^+^ [M + H]^+^: 504.2857; found: 504.2863. Purity: 98.680% by HPLC (A: H_2_O; B: methanol, graded: 95–100%), t_R_ 2.427 min, λ: 292 nm.

*(E)-3-hydroxy-10,13-dimethyl-16-((1-(3,4,5-trimethoxyphenyl)-1H-1,2,3-triazol-4-yl)methylene)-1,3,4,7,8,9,10,11,12,13,15,16-dodecahydro-2H-cyclopenta[a]phenanthren17(14H)-one* (**2u**) Yield, 90%. ^1^H-NMR (300 MHz, CDCl_3_) δ 8.05 (s, 1H, triazole-H), 7.45 (s, 1H,-CO-C=CH), 6.97 (s, 2H, Ar-H), 5.44 (s, 1H, C_6_-H), 3.96 (s, 6H, -OCH_3_), 3.91 (s, 3H, -OCH_3_), 3.24 (dd, *J*_1_ = 18 Hz, *J*_2_ = 3 Hz, 1H, C_3_-H), 2.57–2.47 (m, 1H), 2.35–2.23 (m, 3H), 2.05–1.99 (m, 1H), 1.90–1.83 (m, 1H), 1.78–1.63 (m, 5H), 1.49–1.39 (m, 3H), 1.09–1.00 (m, 8H); ^13^C-NMR (75 MHz, CDCl_3_) δ 209.57, 153.96, 144.72, 141.12, 138.09, 132.47, 123.32, 122.93, 121.88, 121.61, 98.62, 71.55, 61.06, 56.49, 51.33, 49.56, 47.70, 42.20, 37.15, 36.70, 31.55, 31.17, 30.95, 29.68, 20.39, 19.45, 14.28; ESI-HR MS (*m/z*): calcd. for C_31_H_40_N_3_O_5_^+^ [M + H]^+^: 534.2962; found: 534.2955. Purity: 100% by HPLC (A: H_2_O; B: methanol, graded: 80–100%), t_R_ 15.187 min, λ: 292 nm.

*(E)-3-hydroxy-10,13-dimethyl-16-((1-(4-nitrophenyl)-1H-1,2,3-triazol-4-yl)methylene)1,3,4,7,8,9,10,11,12,13,15,16-dodecahydro-2H-cyclopenta[α]phenan- thren-17(14H)-one* (**2v**) Yield 95%. ^1^H-NMR (300 MHz, DMSO-*d*_6_) δ 9.19 (s, 1H, triazole-H), 8.46 (s, 1H,-CO-C=CH), 8.43 (s, 1H, Ar-H), 8.25 (d, *J* = 6 Hz, 2H, Ar-H), 7.24 (s, 1H, Ar-H), 5.31 (s, 1H, C_6_-H), 4.60 (s, 1H, -OH), 2.99 (dd, *J*_1_ = 12 Hz, *J*_2_ = 3 Hz, 1H, C_3_-H), 2.42–2.15 (m, 4H), 1.77–1.66 (m, 7H), 1.49–1.34 (m, 4H), 0.98 (m, 5H), 0.86 (s, 3H); ^13^C-NMR (75 MHz, DMSO-*d*_6_) δ 208.29, 147.28, 144.76, 141.96, 140.95, 138.44, 125.94, 124.55, 121.26, 120.32, 119.74, 70.40, 50.24, 48.90, 47.50, 42.68, 40.30, 40.02, 39.74, 39.47, 39.19, 37.29.36.75, 31.86, 31.58, 31.14, 29.48, 20.42, 19.63, 14.37; ESI-HR MS (*m/z*): calcd. for C_28_H_33_N_4_O_4_^+^ [M + H]^+^: 489.2496; found: 489.2503. Purity: 98.048% by HPLC (A: H_2_O; B: methanol, graded: 80–100%), t_R_ 5.413 min, λ: 292 nm.

*(E)-3-hydroxy-10,13-dimethyl-16-((1-(4-(trifluoromethyl)phenyl)-1H-1,2,3-triazol4-y)methylene)-1,3,4,7,8,9,10,11,12,13,15,16-dodecahydro-2H-cyclopenta[α] phenanthren-17(14H)-one* (**2w**) Yield 93%. ^1^H-NMR (300 MHz, CDCl_3_) δ 8.13 (s, 1H, triazole-H), 7.94 (s, 1H,-CO-C=CH), 7.92 (s, 1H, Ar-H), 7.84 (t, *J* = 3 Hz, 2H, Ar-H), 7.45 (s, 1H, Ar-H), 5.42 (s, 1H, C_6_-H), 3.55 (s, 1H, -OH), 3.22 (dd, *J*_1_ = 9 Hz, *J*_2_ = 3 Hz, 1H, C_3_-H), 2.51 (t, *J* = 9 Hz, 2H, Ar-H), 2.36–2.25 (m, 3H), 2.01–1.69 (m, 7H), 1.52–1.44 (m, 3H), 1.15–1.03 (m, 4H), 0.99 (m, 3H), 0.86 (s, 2H); ^13^C-NMR (75 MHz, CDCl_3_) δ 209.22, 145.32, 141.08, 139.04, 138.74, 131.29, 127.28, 127.23, 122.21, 120.92, 120.54, 119.16, 71.60, 50.36, 49.38, 47.73, 42.23, 37.17, 36.73, 31.59, 31.50, 31.22, 30.98, 29.73, 20.43, 19.47, 14.29; ESI-HR MS (*m/z*): calcd. for C_29_H_33_F_3_N_3_O_2_^+^ [M + H]^+^: 512.2519; found: 512.2509. Purity: 99.465% by HPLC (A: H_2_O; B: methanol, graded: 60–100%), t_R_ 19.600 min, λ: 292 nm.

### 3.3. Biological Evaluation

#### 3.3.1. Cell Cultures and Antiproliferative Assays

The antiproliferative activity of the title compounds against the panel of six different human cancer cell lines, viz. lung (A549), cervix (HeLa), liver (HepG-2), liver (BEL7402), colorectal (HCT116), and breast (MCF-7) cell lines, was evaluated using a standard MTT-based colorimetric assay. All cell lines were obtained from the Key Laboratory of Natural Resources and Functional Molecules of the Changbai Mountain (Yanbian University) and maintained in Dulbecco’s modified Eagle’s medium (DMEM) and RPMI Media 1640 (RPMI1640), supplemented with 10% foetal bovine serum (FBS) at 37 °C in a humidified atmosphere containing 5% CO_2_.

Cells were plated in 96-well plates at appropriate densities to ensure exponential growth throughout the experimental period (9 × 10^3^ cells per well), and then allowed to adhere for 24 h. Cells were then treated for 48 h with each compound. After 48 h of incubation, 10 μL of MTT solution were added to each well to a final concentration of 2 mg·mL^−1^. Plates were then incubated for a further 4 h. After incubation, the MTT solution was removed and 150 μL of DMSO were added to each well for coloration. The plates were shaken vigorously for 10 min at room temperature to ensure complete solubilisation. The optometric density (OD) was read on a microplate reader (EL × 800, BioTek, Highland Park, Winooski, VT, USA) at 492 nm, and the data were subsequently analysed. The percentage of cell growth inhibition was calculated from the following equation:

Inhibitory rate (%) = [1 − (OD_treated_ − OD_blank_)/(OD_control_ − OD_blank_)] × 100.

To obtain the antiproliferative activity of compounds **2a**–**2w**, the compounds were selected in the same way with four serial concentrations (1, 10, 50 and 100 μM) of those compounds. The optometric density (OD) reading was then used to calculate the IC_50_.

#### 3.3.2 Analysis for Cell Cycle by Flow Cytometry

HepG-2 cells were plated in 96-well plates (5.0 × 10^5^ cells per well) and incubated at 37 °C for 12 h. Exponentially growing cells were then incubated with compound **2n** at different concentrations (5, 10 and 20 μM). After 48 h, untreated cells (control) or cells treated with compound **2n** were centrifuged at 1000 rpm (*177* g) for 10 min, and then fixed in 70% ethanol at −20 °C for at least 24 h. The cells were subsequently resuspended in phosphate-buffered saline (PBS) containing 0.1mg mL^−1^ RNase A and 5 μg·mL^−1^ propidium iodide (PI). The cellular DNA content for the cell cycle distribution analysis was measured by flow cytometry using a FACS Calibur flow cytometer with Cell Quest software (Becton-Dickinson, Franklin Lakes, NJ, USA), plotting at least 30,000 events per sample. The percentage of cells in the G1, S and G2 phases of the cell cycle were determined using the ModFit LT V4.0 software package (Verity Software, Topsham, ME, USA).

#### 3.3.3. Analysis for Apoptosis by Flow Cytometry

Apoptosis was detected using an Apoptosis Detection Kit (Invitrogen, Eugene, OR, USA). In brief, cells were cultured in 96-well plates (5.0 × 10^5^ cells per well) and incubated at 37 °C for 12 h. Cells with exponential growth were then incubated with compound **2n** at different concentrations (5, 10 and 20 μM). Following 48 h of incubation, the cells were collected, washed twice with PBS and once with 1binding buffer, and then stained with 5 μM of annexin V-FITC and 2.5 μM of PI (5 mg·mL^−1^) in 1 × binding buffer for 30 min at 20 °C in the dark. Apoptotic cells were enumerated using a FACSCalibur flow cytometer with Cell Quest software (Becton–Dickinso, Franklin Lakes, NJ, USA).

## 4. Conclusions

In summary, we designed and synthesised one series of DHEA derivatives (compounds **2a**–**2w**) and evaluated their antiproliferative effects against six cancer cell lines (A549, Hela, HepG-2, BEL7402, MCF-7, and HCT116). Several of the target compounds exhibited potent inhibitory activity in vitro and the antiproliferative activity of these compounds was screened via the MTT assay. In particular, compound **2n** exhibited excellent inhibitory activity against HepG-2 cells, with an IC_50_ value of 9.10 µM. Moreover, it showed high inhibitory activities against MCF-7, with IC_50_ values of 9.18 µM. The results of experiments on cell cycle arrest and apoptosis induced by compound **2n** suggested that it induced apoptosis in HepG-2 cells. Therefore, the modifications to the C_16_ position of DHEA in the present study were helpful in improving its antiproliferative activity.

## Figures and Tables

**Table 1:** The antiproliferative activity of compounds **2a**–**2w** (% Growth Inhibition at 100 μM). DHEA: Dehydroepiandrosterone, NA: antiproliferative activity <5%.

Compound	R	% Growth Inhibition at 100 μM						
		A549	Hela	HepG-2	BEL7402	HCT116	MCF-7	L02
**DHEA**	-	24.8	15.7	10.9	46.5	47.3	17.1	44.6
**2a**	H	30.3	NA	10.2	12.9	11.1	NA	22.3
**2b**	2-F	NA	NA	NA	NA	NA	NA	NA
**2**	3-F	60.5	55.2	78.8	66.7	78.3	31.2	79.3
**2d**	4-F	68.8	78.9	65.6	60.4	84.9	83.0	80.3
**2e**	2-Cl	NA	32.8	NA	10.5	23.0	37.3	12.4
**2f**	3-Cl	11.9	9.2	51.7	36.5	41.9	41.0	60.2
**2g**	4-Cl	69.8	62.8	32.5	66.9	78.7	39.9	40.9
**2h**	3,4-Cl_2_	70.7	68.7	48.2	70.1	74.4	80.0	67.9
**2i**	2-Br	NA	17.0	NA	NA	20.2	NA	NA
**2j**	3-Br	NA	20.5	10.8	37.6	42.0	42.1	43.8
**2k**	4-Br	47.8	30.7	66.4	NA	NA	45.5	11.1
**2l**	2-I	NA	NA	NA	NA	NA	NA	NA
**2m**	3-I	NA	NA	NA	14.1	7.6	NA	NA
**2n**	4-I	46.8	17.3	83.0	NA	65.5	81.3	34.2
**2o**	3-CH_3_	NA	12.7	12.8	30.5	40.9	11.2	40.1
**2p**	4-CH_3_	63.1	64.6	76.0	23.7	76.5	21.7	67.1
**2q**	2-OCH_3_	7.6	NA	NA	10.0	14.3	34.6	NA
**2r**	3-OCH_3_	NA	NA	NA	NA	NA	NA	16.9
**2s**	4-OCH_3_	44.0	14.9	42.3	38.4	70.7	58.1	58.5
**2t**	3,4-(OCH_3_)_2_	NA	NA	NA	NA	NA	NA	9.4
**2u**	3,4,5-(OCH_3_)_3_	40.3	60.5	59.1	51.2	59.2	36.3	36.4
**2v**	4-NO_2_	NA	NA	12.4	13.2	10.2	NA	5.5
**2w**	4-CF_3_	75.2	44.1	48.3	62.2	75.0	41.3	57.6

**Table 2:** IC_50_ **values** (μM) of some active compounds.

Compound	R	IC_50_ Values (μM) ^a^						
		A549	Hela	HepG-2	BEL7402	HCT116	MCF-7	L02
**2c**	3-F	51.64	83.52	11.07	68.86	26.32	>100	83.37
**2d**	4-F	15.80	13.21	36.19	40.41	33.25	17.75	27.75
**2g**	4-Cl	37.38	57.3	>100	58.22	23.37	>100	>100
**2h**	3,4-Cl_2_	17.46	15.11	>100	15.96	11.86	14.93	26.36
**2k**	4-Br	>100	>100	37.11	>100	>100	>100	>100
**2n**	4-I	>100	>100	9.10	>100	31.04	9.18	>100
**2p**	4-CH_3_	56.72	62.42	18.94	>100	44.07	>100	62.23
**2s**	4-OCH_3_	>100	>100	>100	>100	56.91	69.4	81.08
**2w**	4-CF_3_	72.55	>100	>100	94.03	22.65	>100	93.69
**5-FU**		23.65	34.61	10.59	21.30	24.80	28.11	19.12

^a^ IC_50_: concentration that inhibits 50% of cell growth.

**Table 3:** In vitro antiproliferative activities of compounds **2c**, **2d**, and **2n** against normal cell line (L02).

Compounds	R	(IC_50_, μM)	Selectivity Index ^a^					
		L02	Hela	HepG-2	BEL7402	HCT116	MCF-7	A549
**2c**	3-F	83.37	0.99	7.53	1.21	3.17	- ^b^	1.61
**2d**	4-F	27.75	2.10	0.77	0.69	0.83	1.56	1.76
**2n**	4-I	>100	-	>10.99	-	>3.22	>10.89	-
**5-FU**	-	19.12	0.55	1.80	0.90	0.65	1.47	0.81

^a^ SI: selective index (IC_50_ on normal cells/IC_50_ on tumour cells). ^b^ not calculated.

## Supplementary Materials

The Supplementary materials are available online, ^1^H and ^13^C-NMR spectra of these compounds are available in the supplementary materials.
