# Supplementary material for: Design and Synthesis of Novel Dehydroepiandrosterone Analogues as Potent Antiproliferative Agents
Source: Molecules. 2018 Sep 3;23(9):2243. doi: 10.3390/molecules23092243 (PMC6225165; doi:10.3390/molecules23092243)
Supplement: Supplementary file 1 [file molecules-23-02243-s001.pdf]

Design and synthesis of novel dehydroepiandrosterone analogues as potent anticancer agents

Xing Huang, Qing-Kun Shen, Hong-Jian Zhang, Jia-Li Li, Hai-Ming Zhang, Yu-Shun Tian* and Zhe-Shan Quan*

Key Laboratory of Natural Resources and Functional Molecules of the Changbai Mountain, Affiliated Ministry of Education, College of Pharmacy, Yanbian University, Yanji, Jilin, 133002, China.

***Corresponding author: Tel: + 86 433 243-6020; Fax: + 86 433 243-6020.**

E-mail: zsquan@ybu.edu.cn (Z. S. Quan).

***Corresponding author: Tel: + 86 433 243-6028; Fax: + 86 433 243-5026.**

E-mail: ystian@ybu.edu.cn (Y. S. Tian).

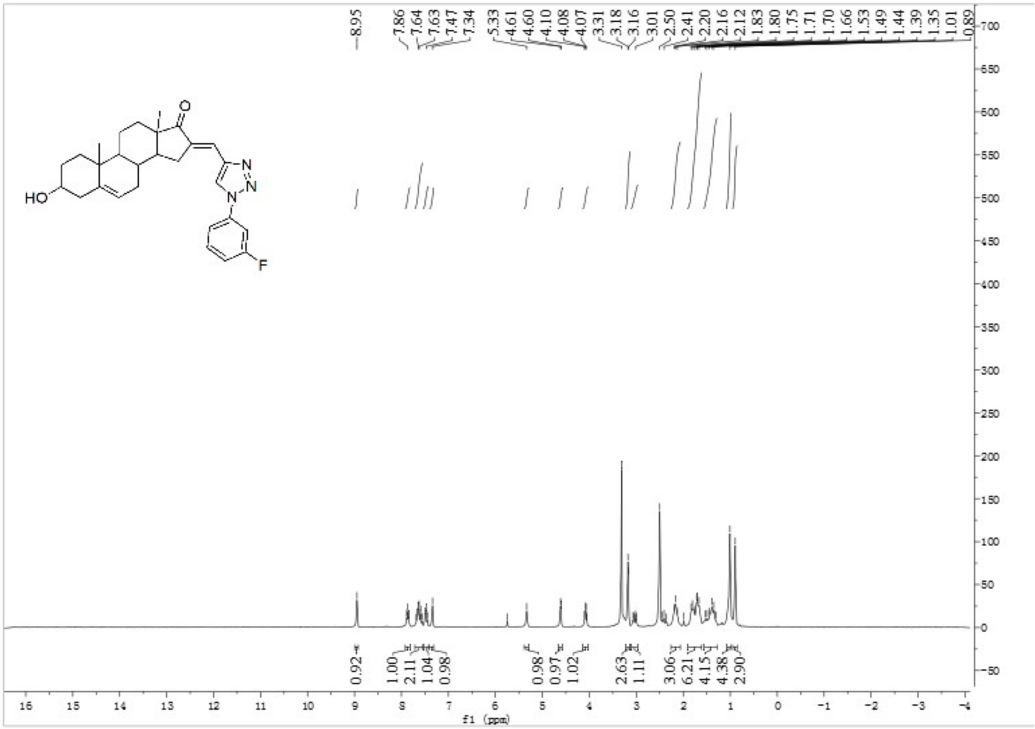

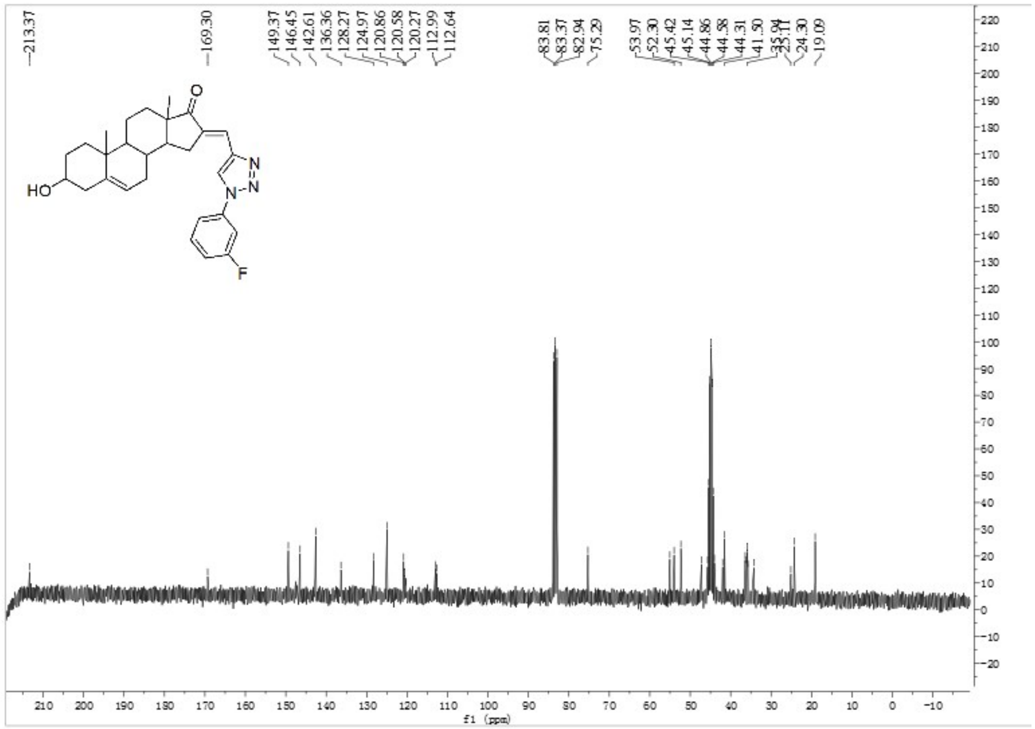

^{1}H-NMR,13C-NMR:(*E*)-16-((1-(3-fluorophenyl)-1*H*-1,2,3-triazol-4-yl)methylene)-3-hydroxy-10,13-di-methyl-1,3,4,7,8,9,10,11,12,13,15,16-dodecahydro-2*H*-cyclopenta[a]phenanthren-17(14*H*)-one (2c)

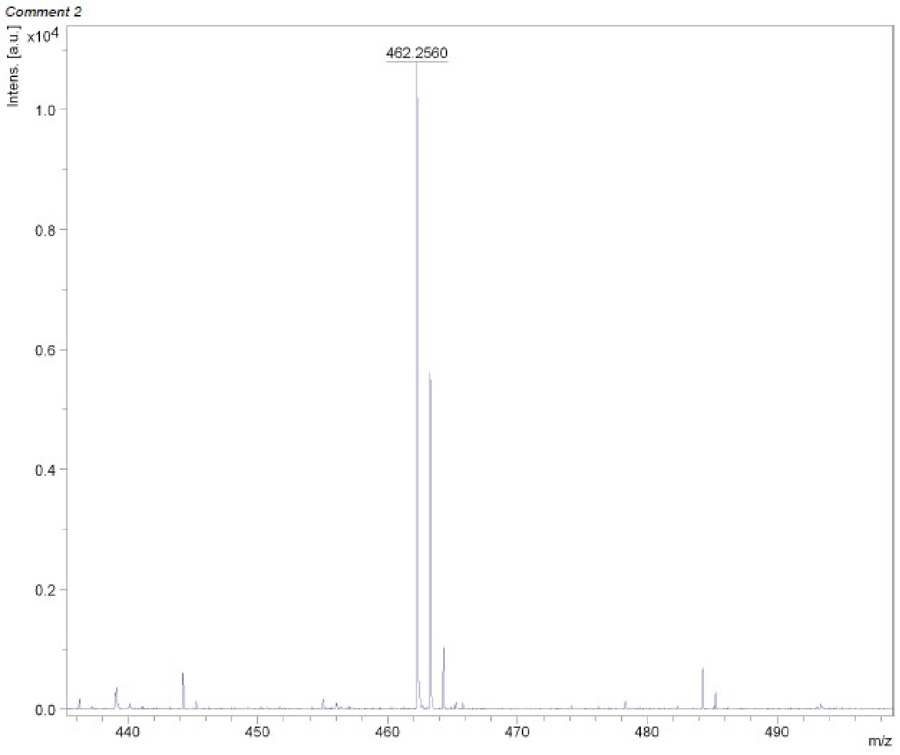

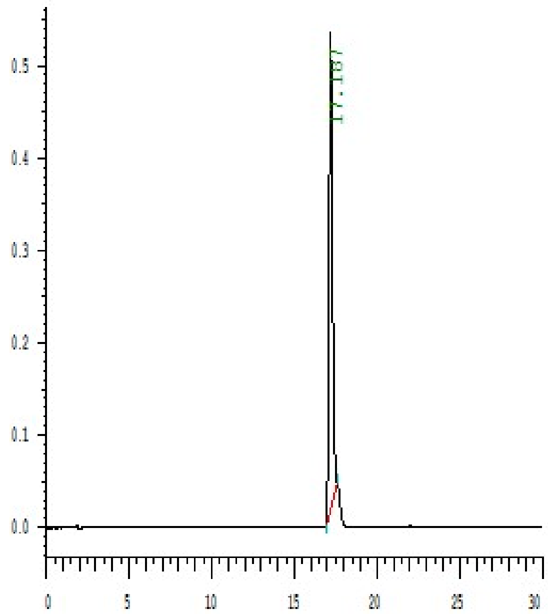

HRMS,HPLC:(*E*)-16-((1-(3-fluorophenyl)-1*H*-1,2,3-triazol-4-yl)methylene)-3-hydroxy-10,13-di-methyl-1,3,4,7,8,9,10,11,12,13,15,16-dodecahydro-2*H*-cyclopenta[a]phenanthren-17(14*H*)-one (2c)

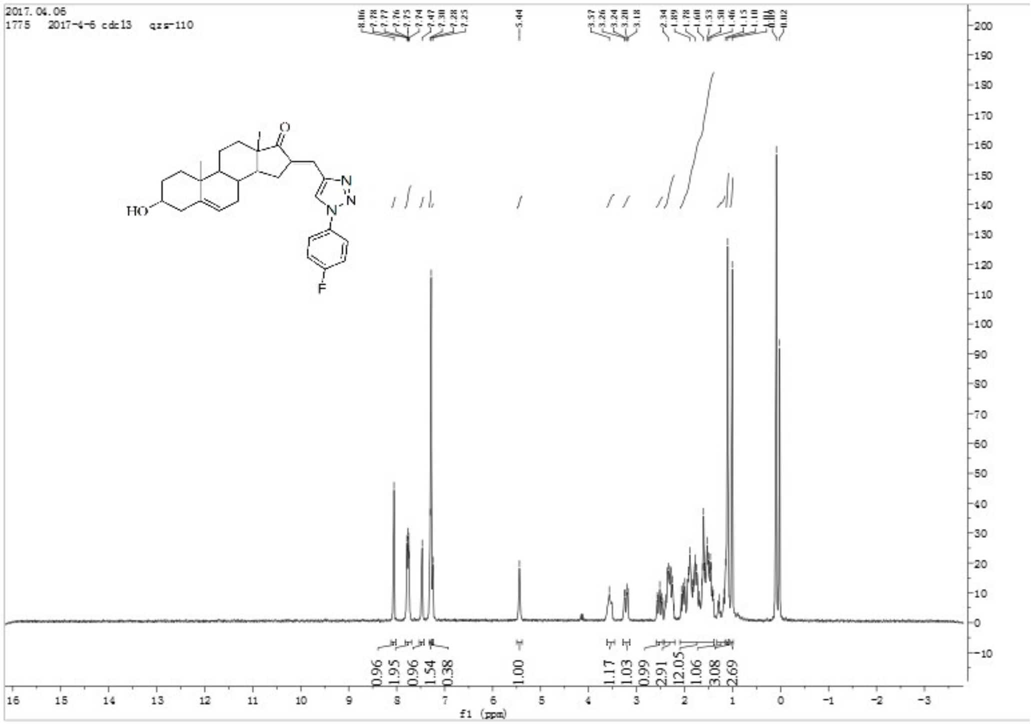

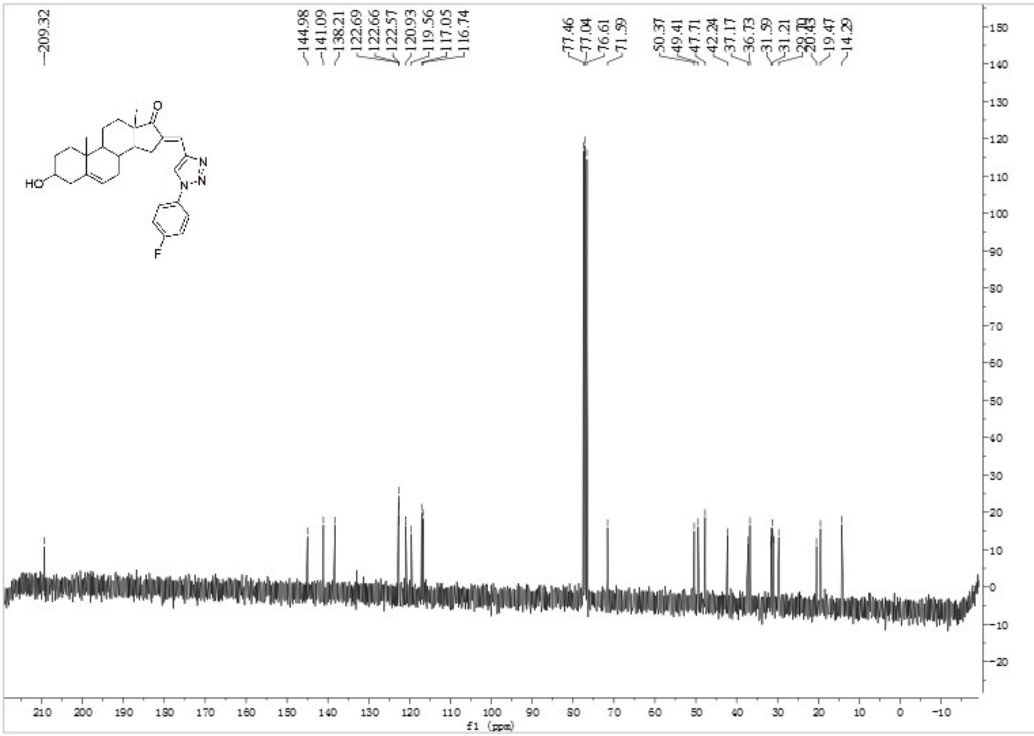

^{1}H-NMR,13C-NMR:(*E*)-16-((1-(4-fluorophenyl)-1*H*-1,2,3-triazol-4-yl)methylene)-3-hydroxy-10,13-di-methyl-1,3,4,7,8,9,10,11,12,13,15,16-dodecahydro-2*H*-cyclopenta[a]phenanthren-17(14*H*)-one (2d)

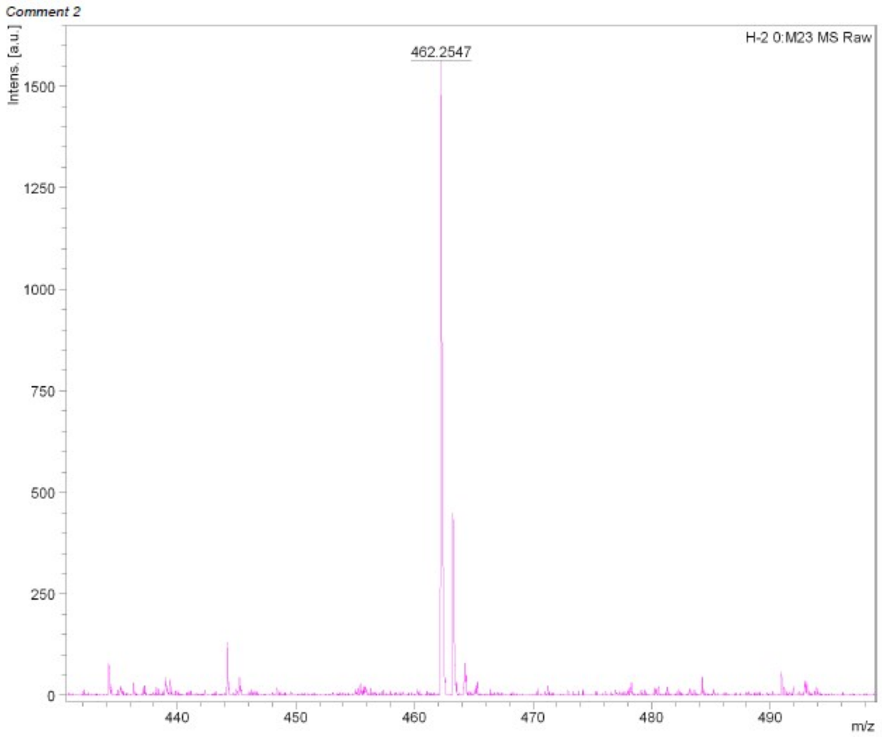

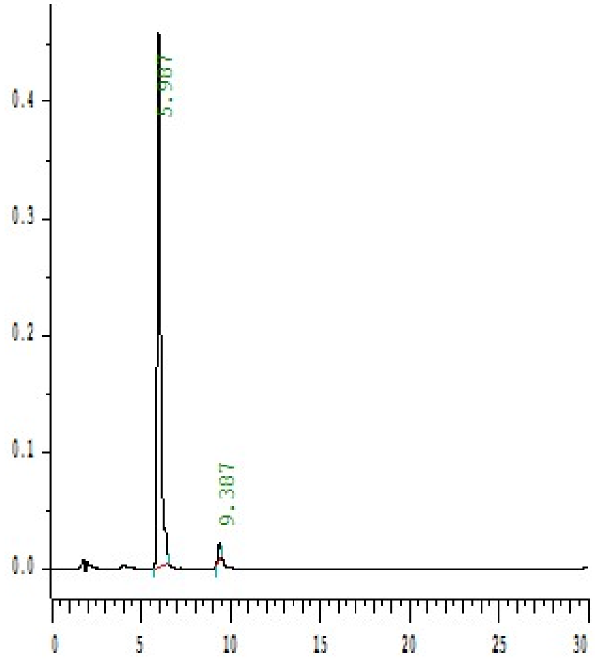

HRMS,HPLC:(*E*)-16-((1-(4-fluorophenyl)-1*H*-1,2,3-triazol-4-yl)methylene)-3-hydroxy-10,13-di-methyl-1,3,4,7,8,9,10,11,12,13,15,16-dodecahydro-2*H*-cyclopenta[a]phenanthren-17(14*H*)-one (2d)

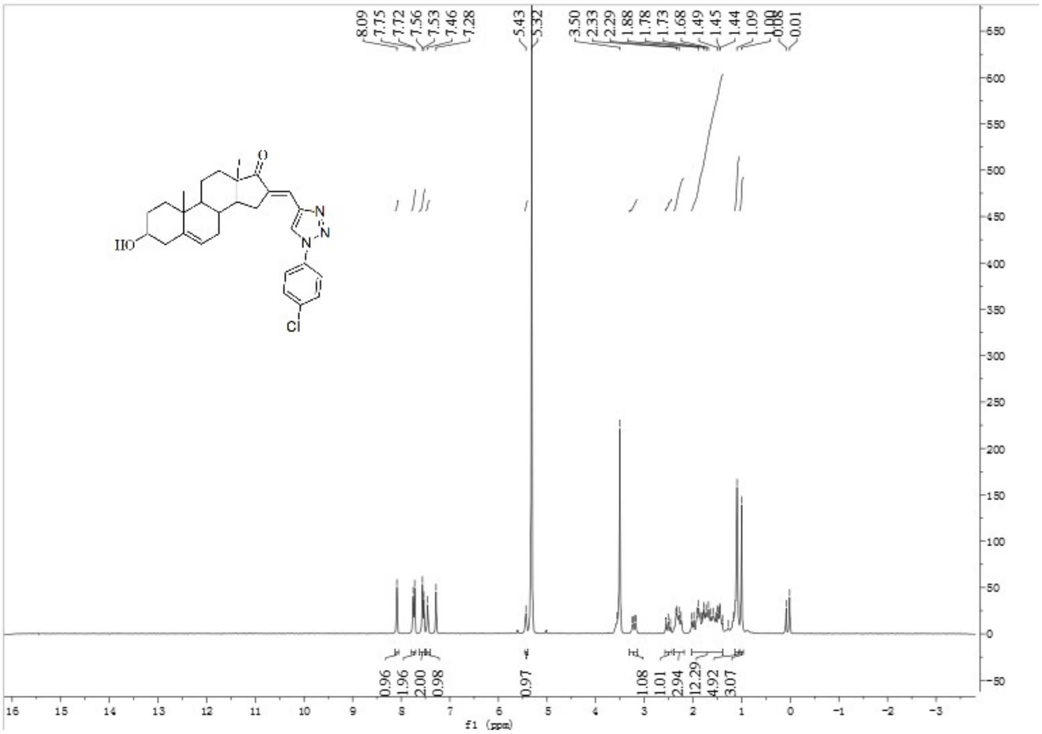

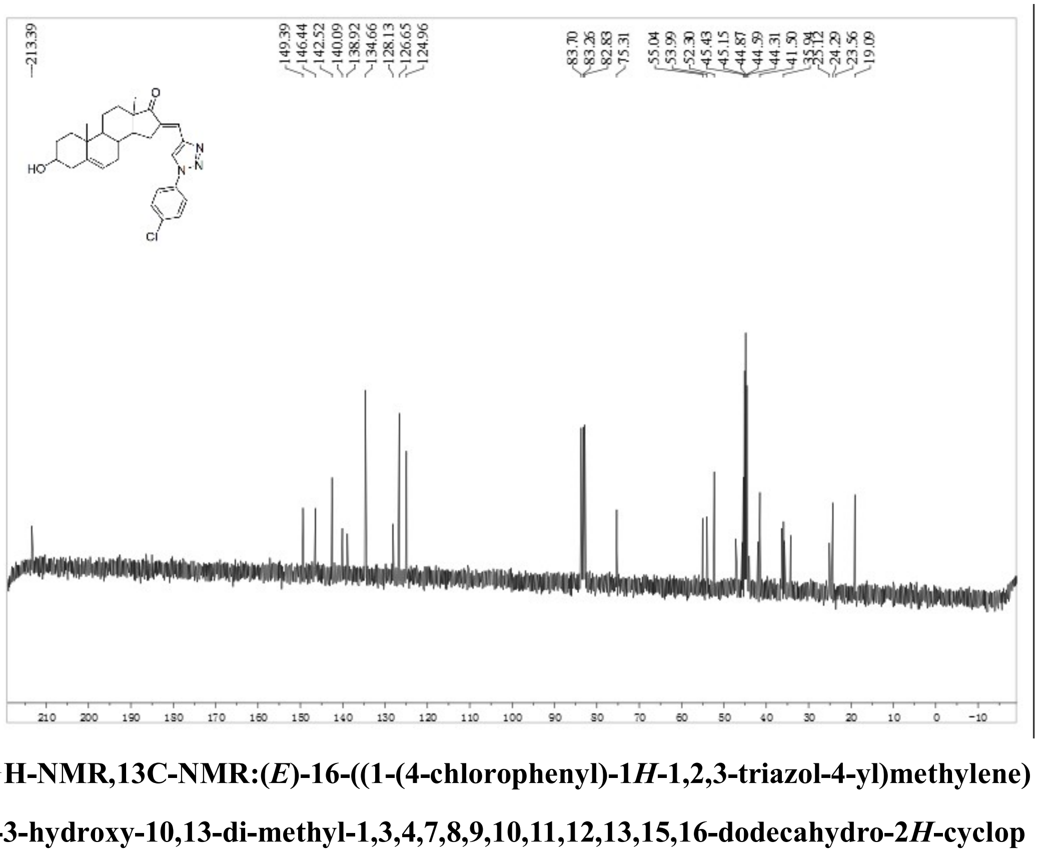

^{1}H-NMR,13C-NMR:(*E*)-16-((1-(4-chlorophenyl)-1*H*-1,2,3-triazol-4-yl)methylene)-3-hydroxy-10,13-di-methyl-1,3,4,7,8,9,10,11,12,13,15,16-dodecahydro-2*H*-cyclopenta[a]phenanthren-17(14*H*)-one (2g)

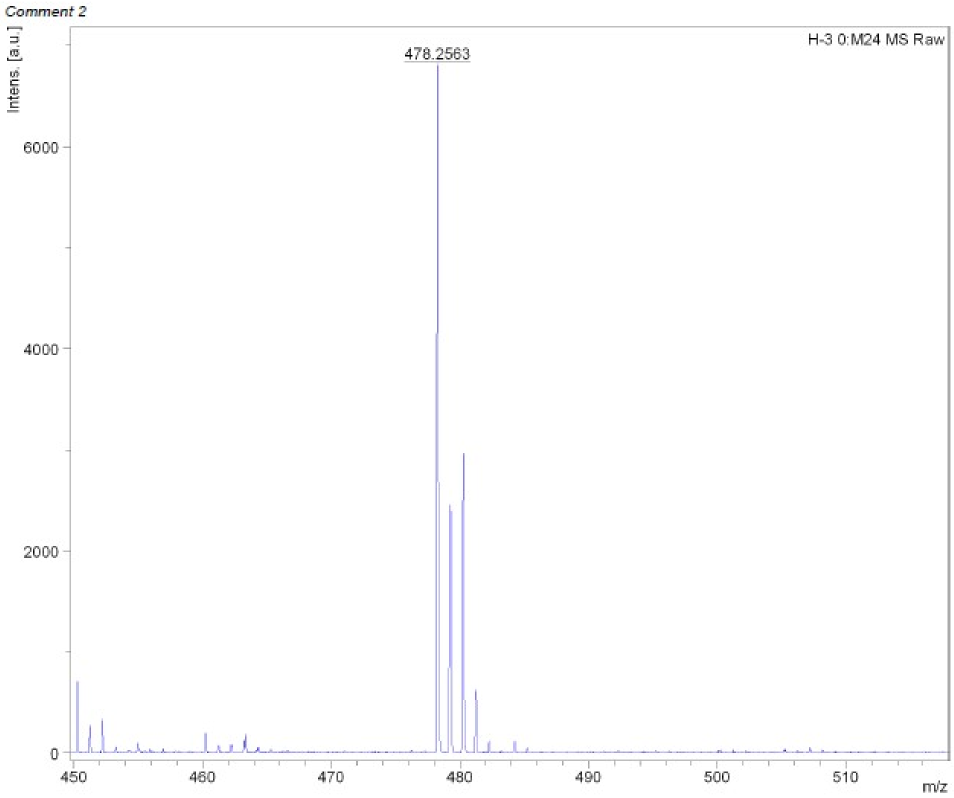

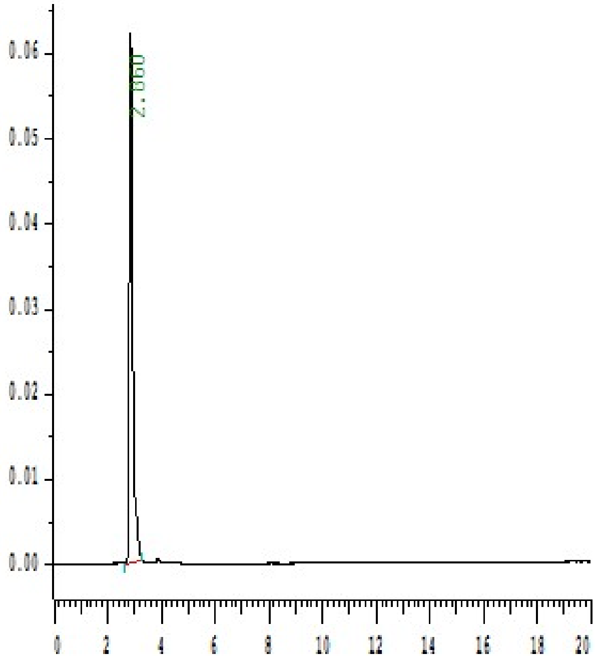

HRMS,HPLC:(*E*)-16-((1-(4-chlorophenyl)-1*H*-1,2,3-triazol-4-yl)methylene)-3-hydroxy-10,13-di-methyl-1,3,4,7,8,9,10,11,12,13,15,16-dodecahydro-2*H*-cyclopenta[a]phenanthren-17(14*H*)-one (2g)

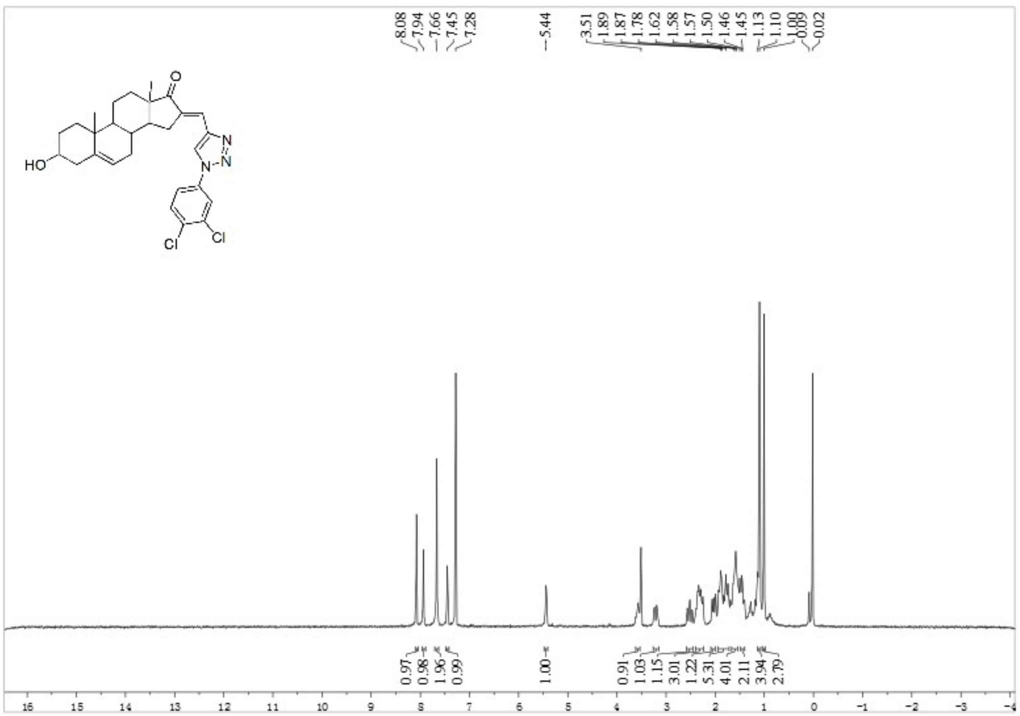

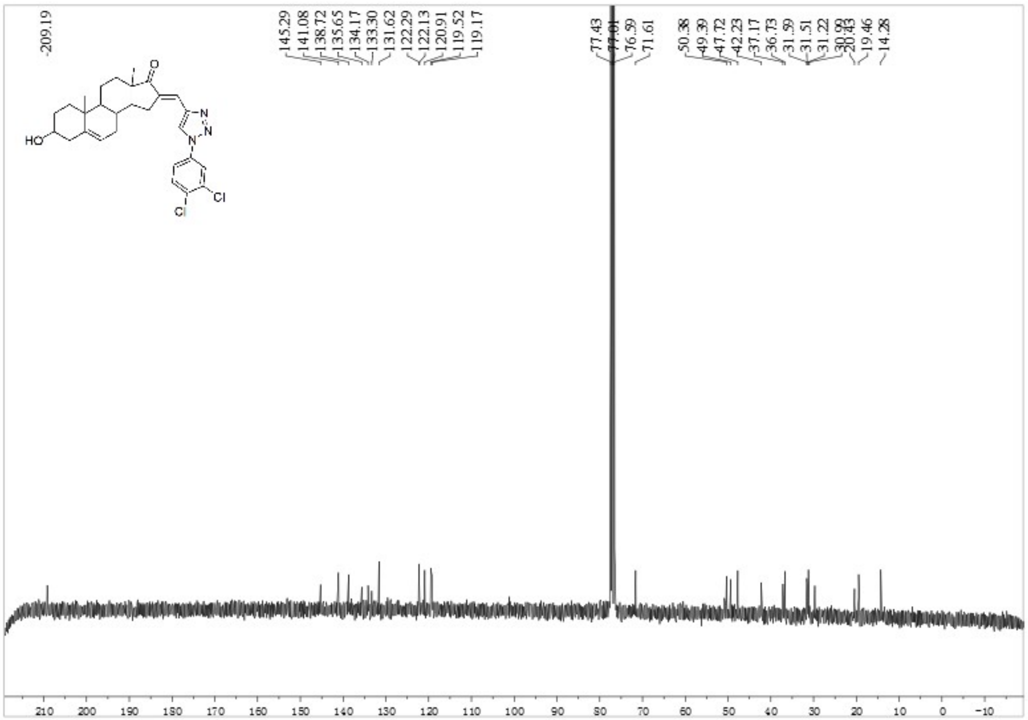

^{1}H-NMR,13C-NMR:(*E*)-16-((1-(3,4-dichlorophenyl)-1*H*-1,2,3-triazol-4-yl)methylene)-3-hydroxy-10,13dimethyl-1,3,4,7,8,9,10,11,12,13,15,16-dodecahydro-2*H*-cyclopenta[a]phenanthren-17(14*H*)-one (2h)

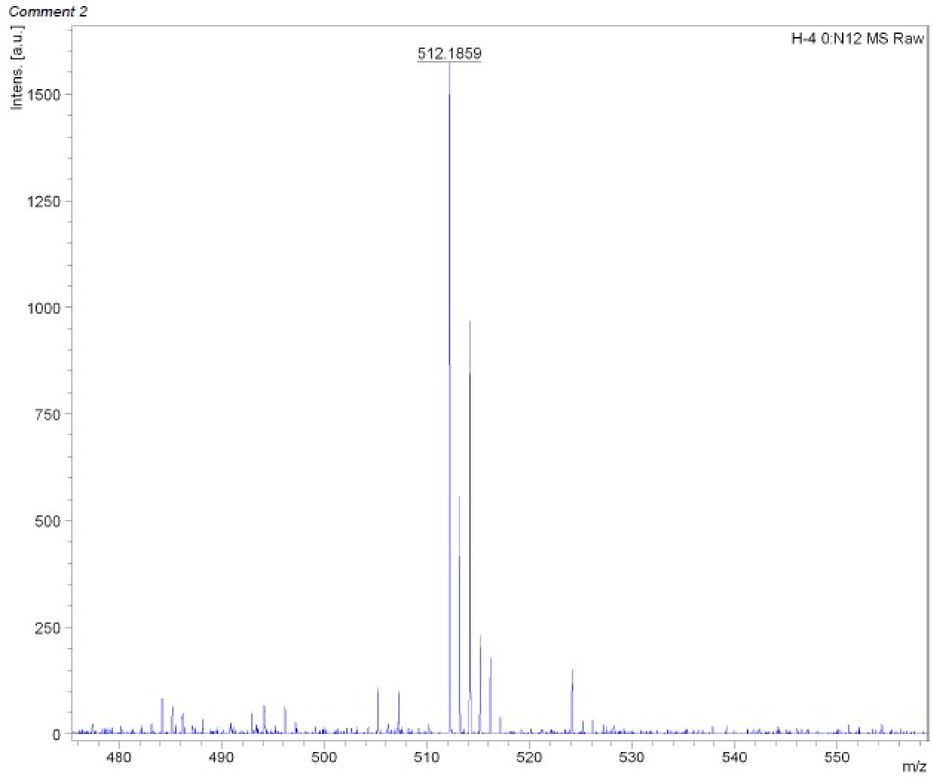

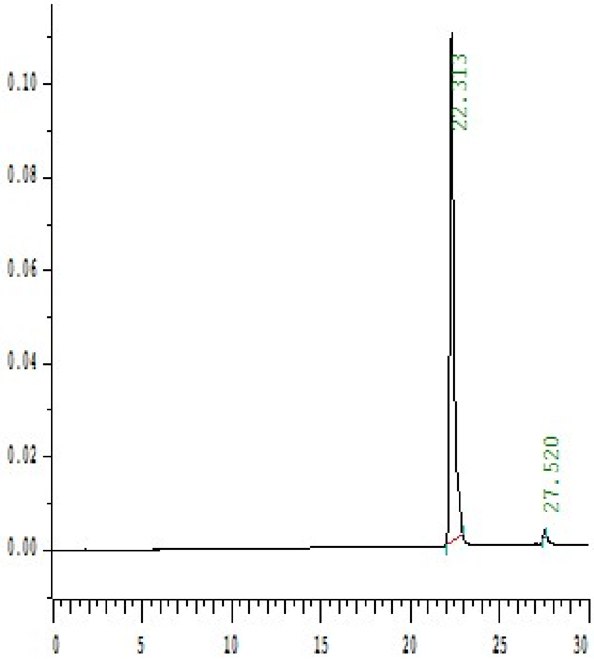

HRMS,HPLC:(*E*)-16-((1-(3,4-dichlorophenyl)-1*H*-1,2,3-triazol-4-yl)methylene)-3-hydroxy-10,13dimethyl-1,3,4,7,8,9,10,11,12,13,15,16-dodecahydro-2*H*-cyclopenta[a]phenanthren-17(14*H*)-one (2h)

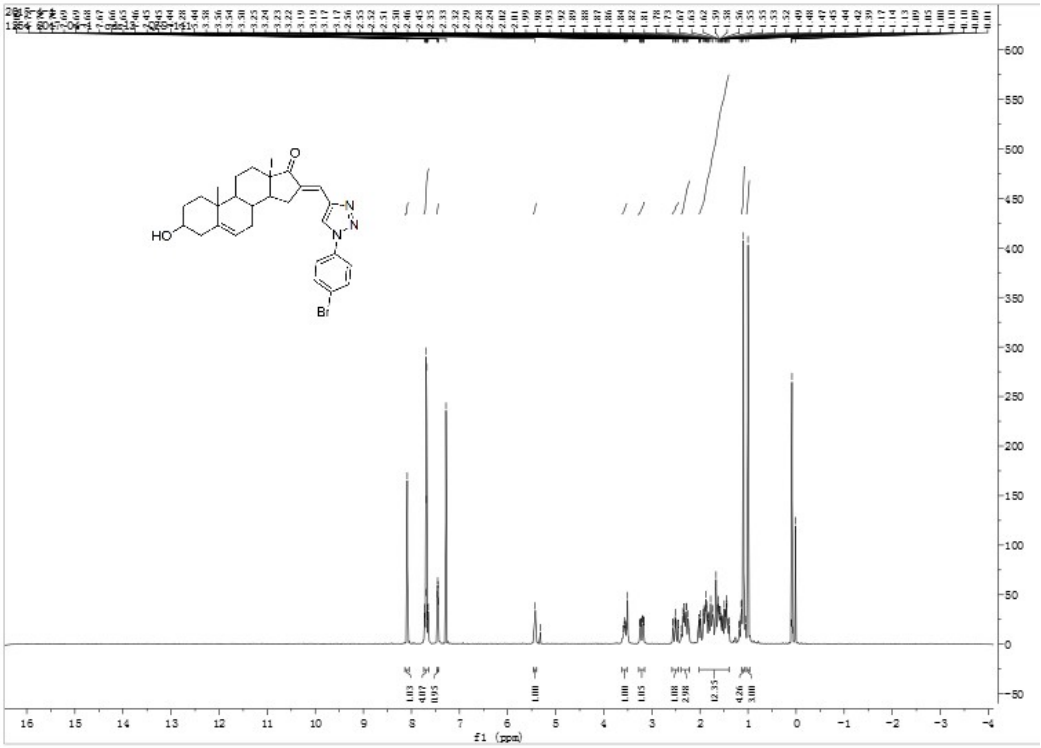

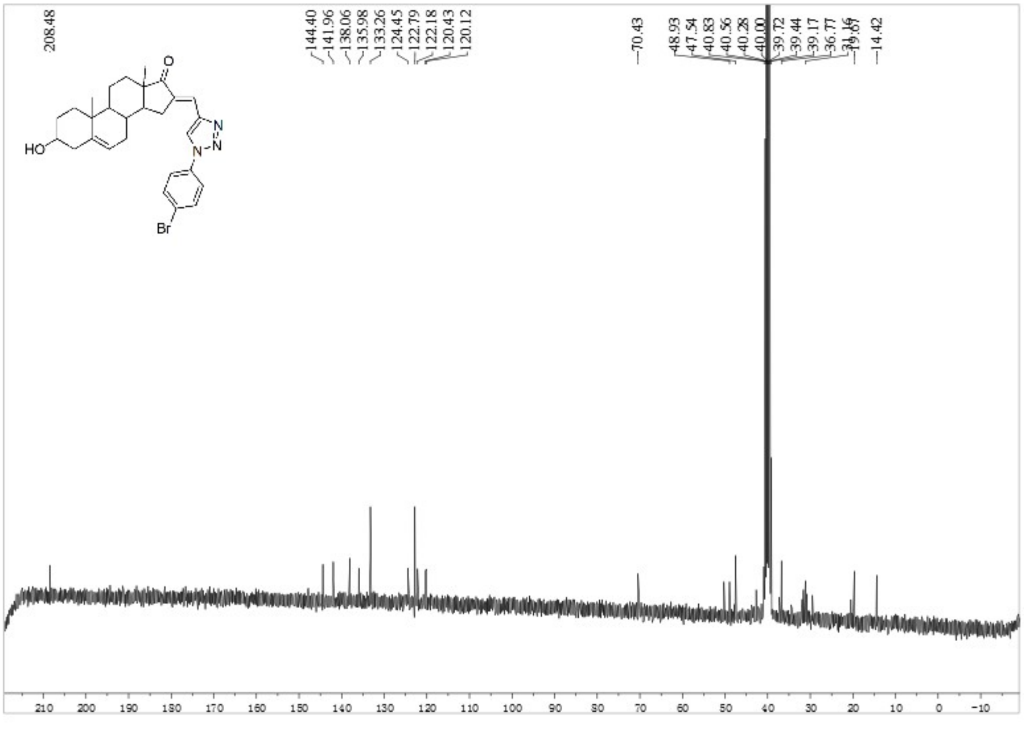

^{1}H-NMR,13C-NMR:(*E*)-16-((1-(4-bromophenyl)-1*H*-1,2,3-triazol-4-yl)methylene)-3-hydroxy-10,13-di-methyl-1,3,4,7,8,9,10,11,12,13,15,16-dodecahydro-2*H*-cyclopenta[a]phenanthren-17(14*H*)-one(2k)

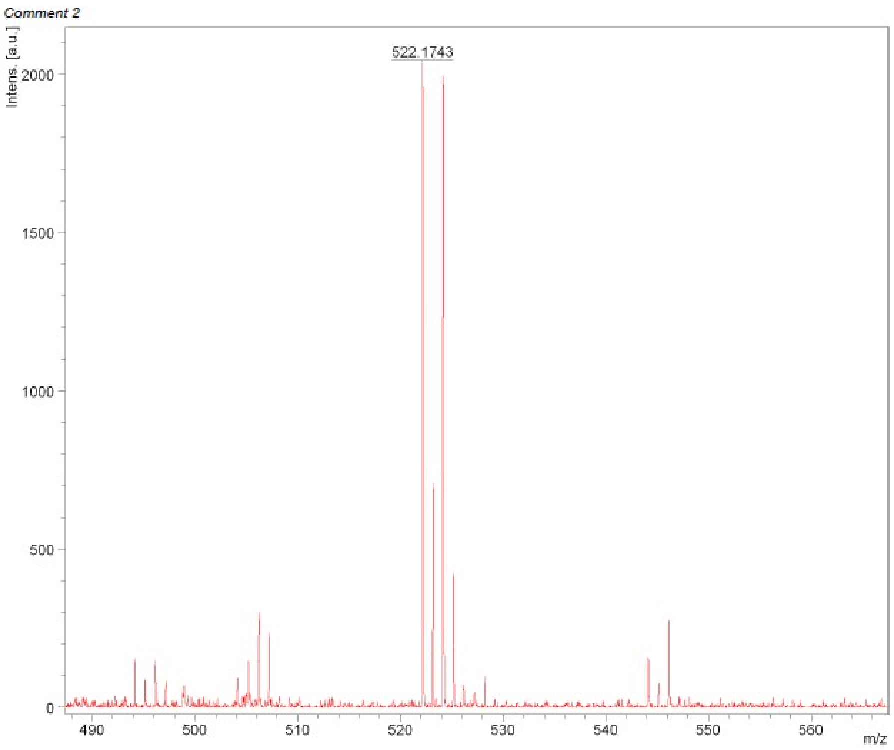

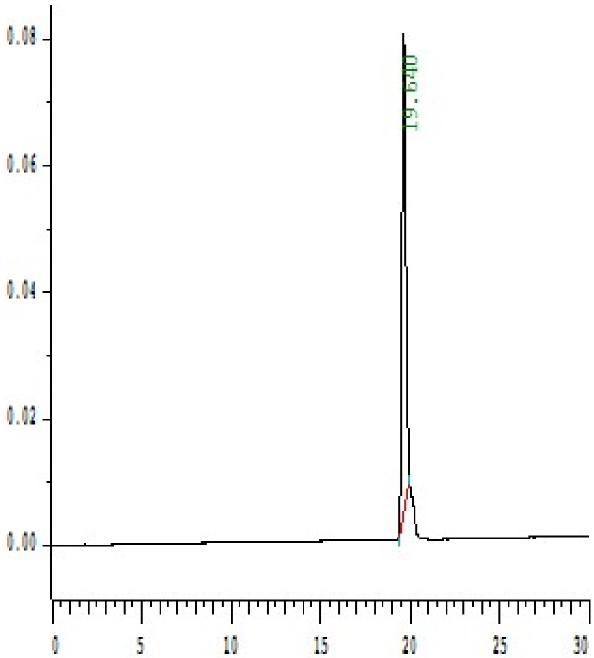

HRMS,HPLC:(*E*)-16-((1-(4-bromophenyl)-1*H*-1,2,3-triazol-4-yl)methylene)-3-hydroxy-10,13-di-methyl-1,3,4,7,8,9,10,11,12,13,15,16-dodecahydro-2*H*-cyclopenta[a]phenanthren-17(14*H*)-one(2k)

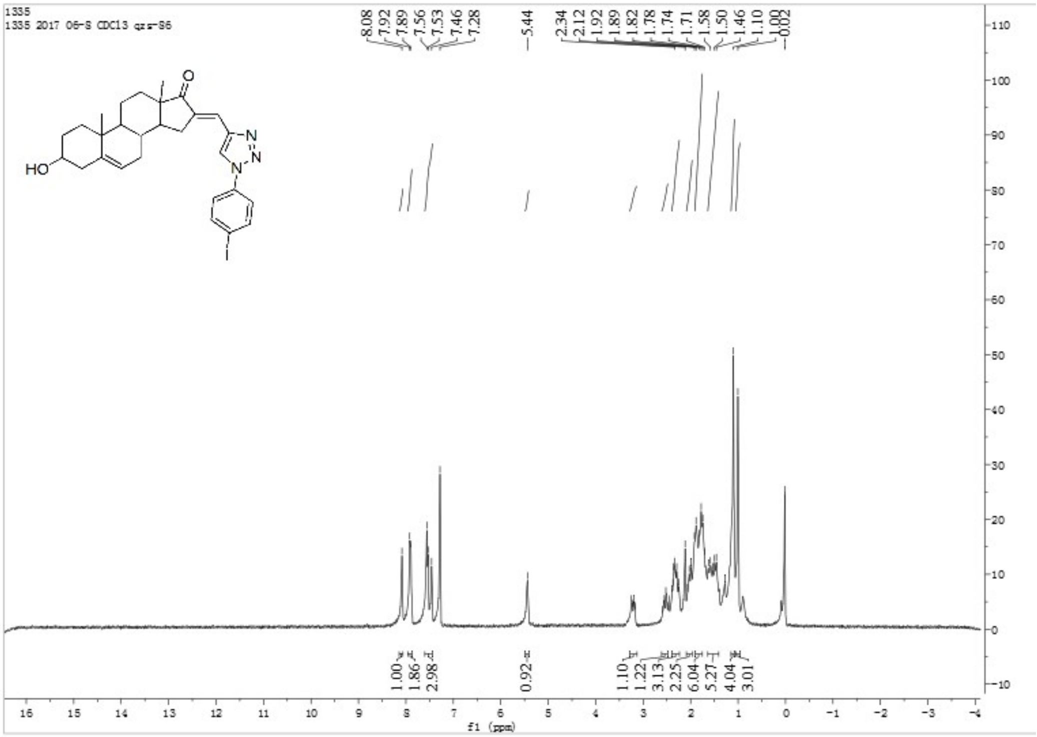

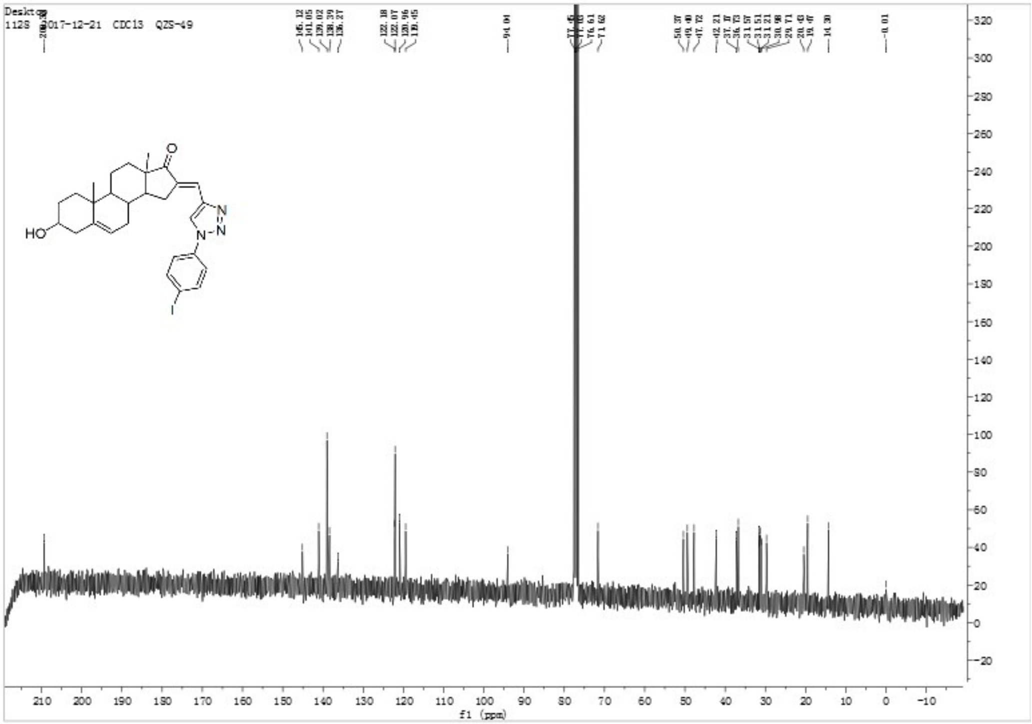

^{1}H-NMR,13C-NMR:(*E*)-3-hydroxy-16-((1-(4-iodophenyl)-1*H*-1,2,3-triazol-4-yl)methylene)-10,13-dim-ethyl-1,3,4,7,8,9,10,11,12,13,15,16-dodecahydro-2*H*-cyclopenta[a]phenanthren-17(14*H*)-one (2n)

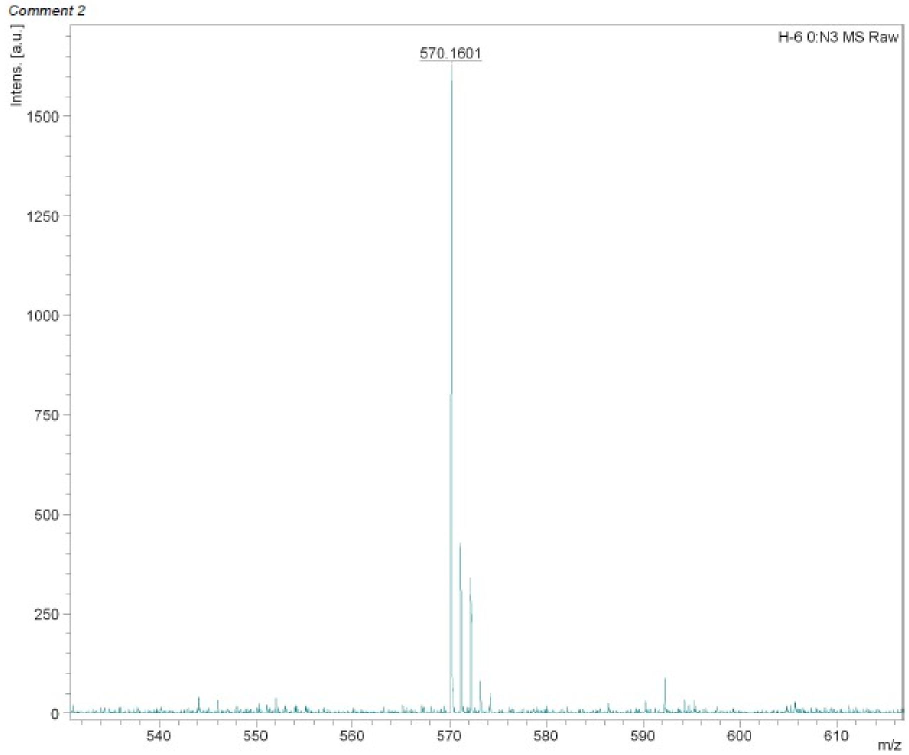

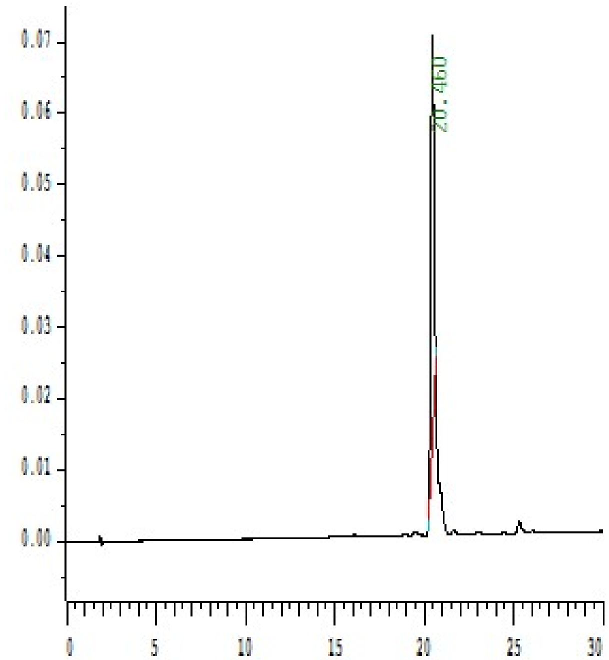

HRMS,HPLC:(*E*)-3-hydroxy-16-((1-(4-iodophenyl)-1*H*-1,2,3-triazol-4-yl)methylene)-10,13-dim-ethyl-1,3,4,7,8,9,10,11,12,13,15,16-dodecahydro-2*H*-cyclopenta[a]phenanthren-17(14*H*)-one (2n)

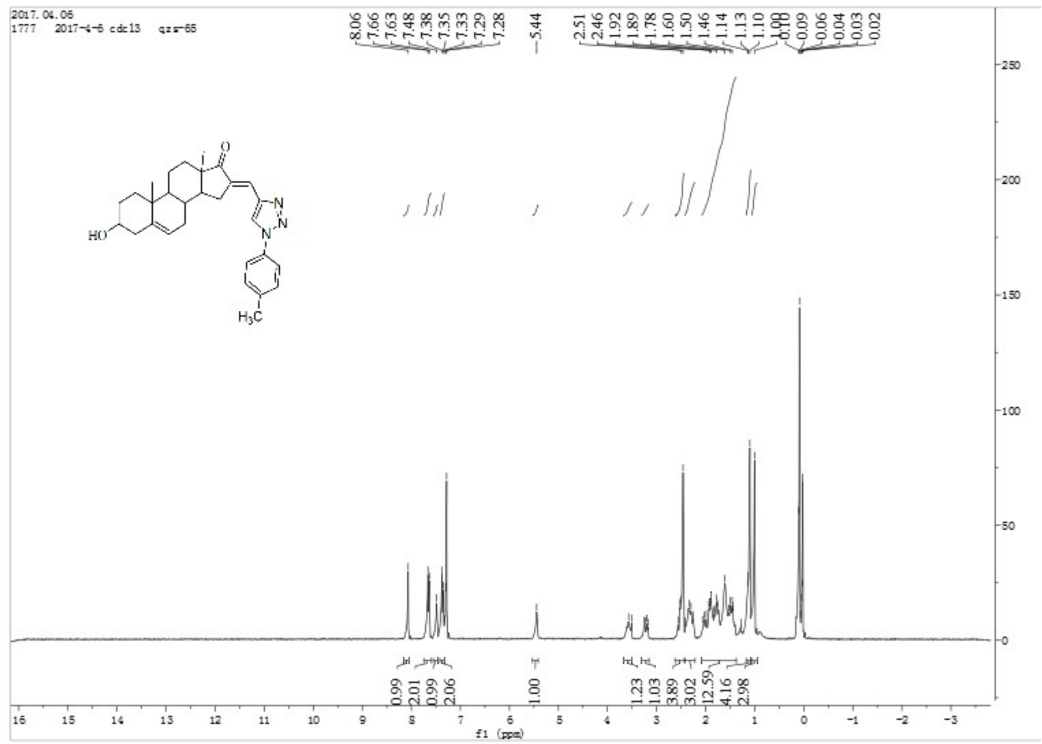

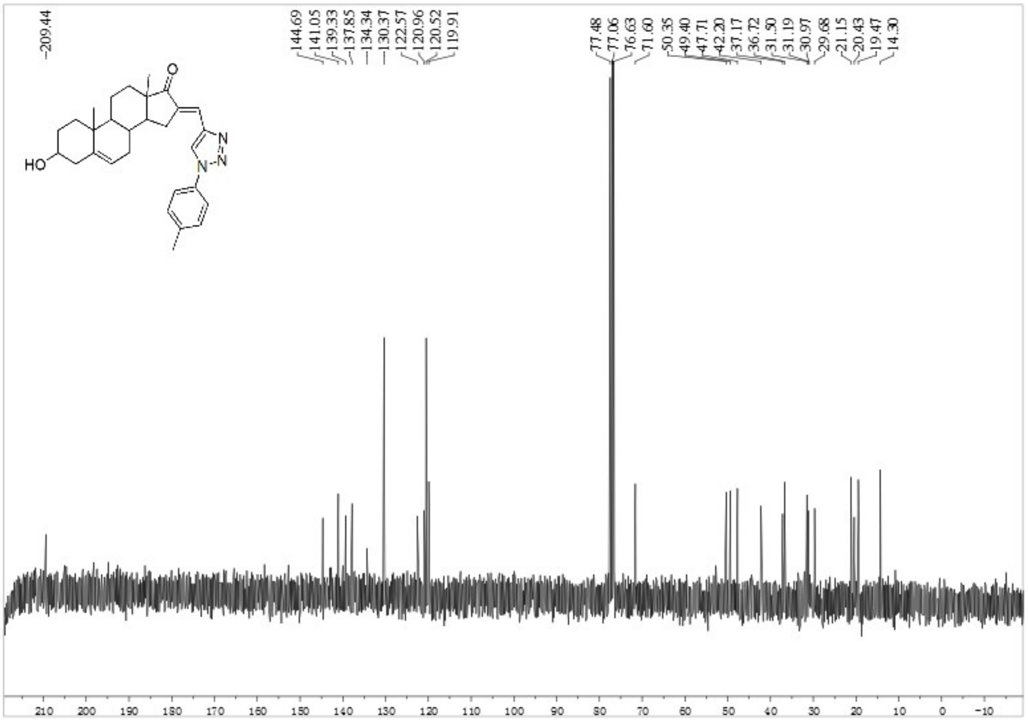

^{1}H-NMR,13C-NMR:(*E*)-3-hydroxy-10,13-dimethyl-16-((1-p-tolyl-1*H*-1,2,3-triazol-4-yl)methylene)-1,3,4,7,8,9,10,11,12,13,15,16-dodecahydro-2*H*-cyclopenta[a]phenanthren-17(14*H*)-one (2p)

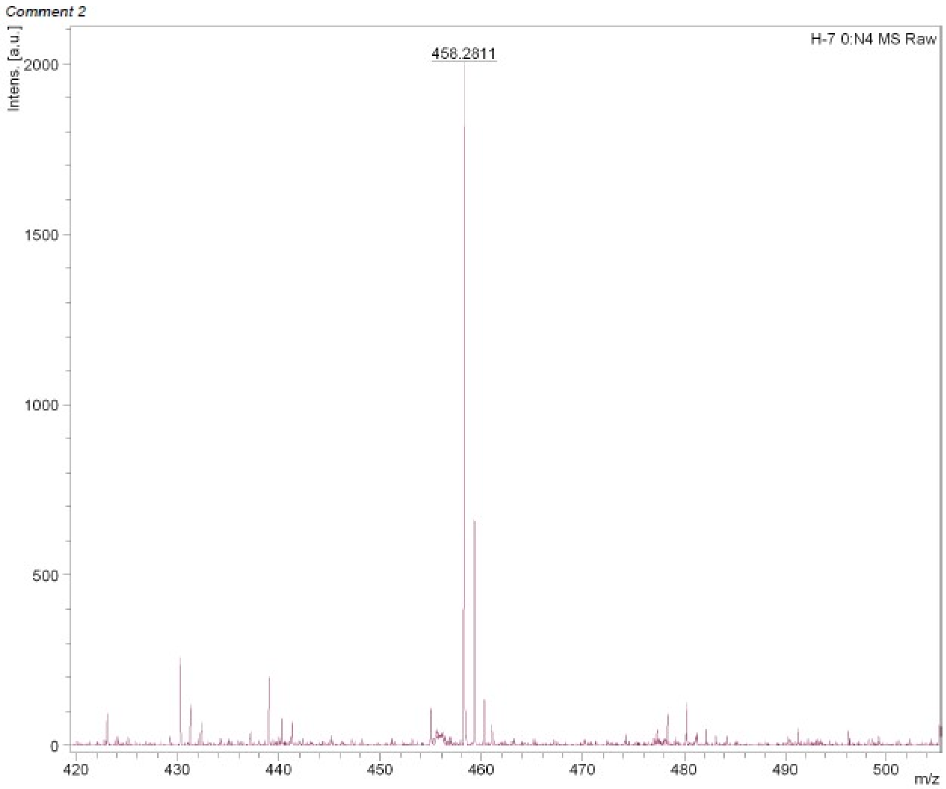

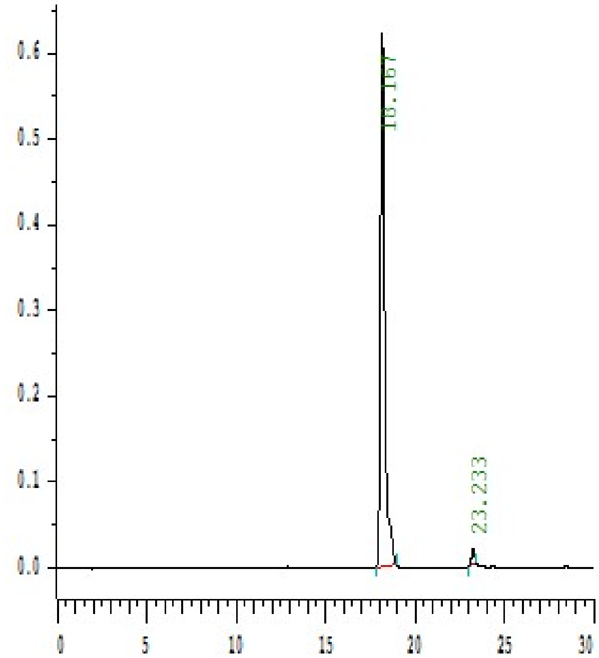

HRMS,HPLC:(*E*)-3-hydroxy-10,13-dimethyl-16-((1-p-tolyl-1*H*-1,2,3-triazol-4-yl)methylene)-1,3,4,7,8,9,10,11,12,13,15,16-dodecahydro-2*H*-cyclopenta[a]phenanthren-17(14*H*)-one (2p)

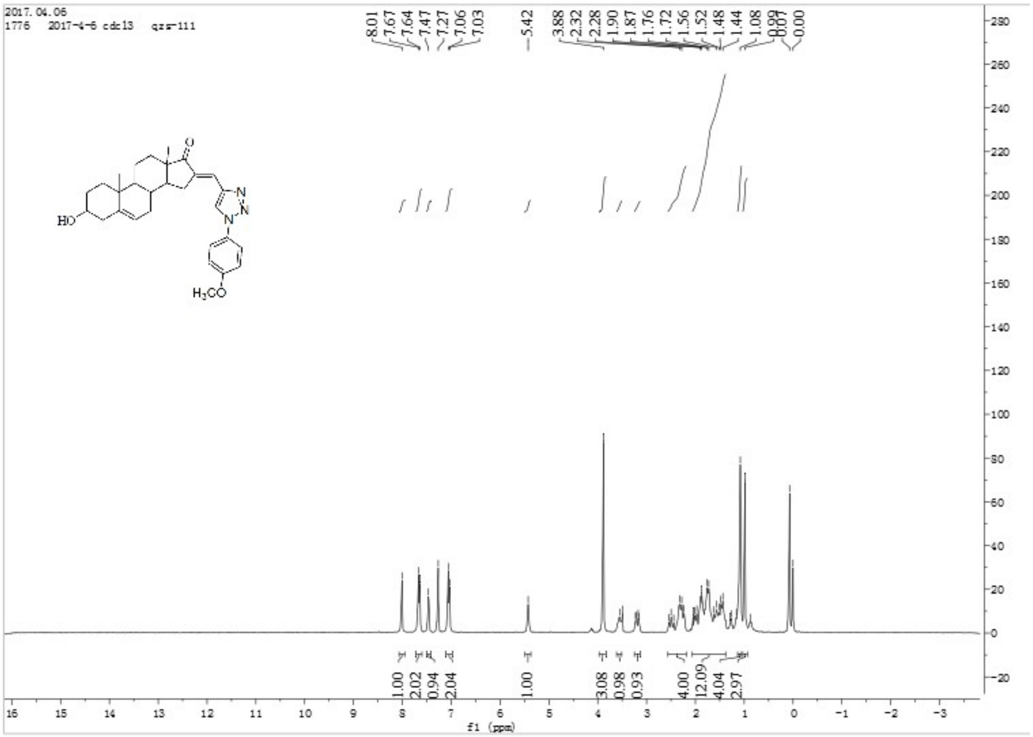

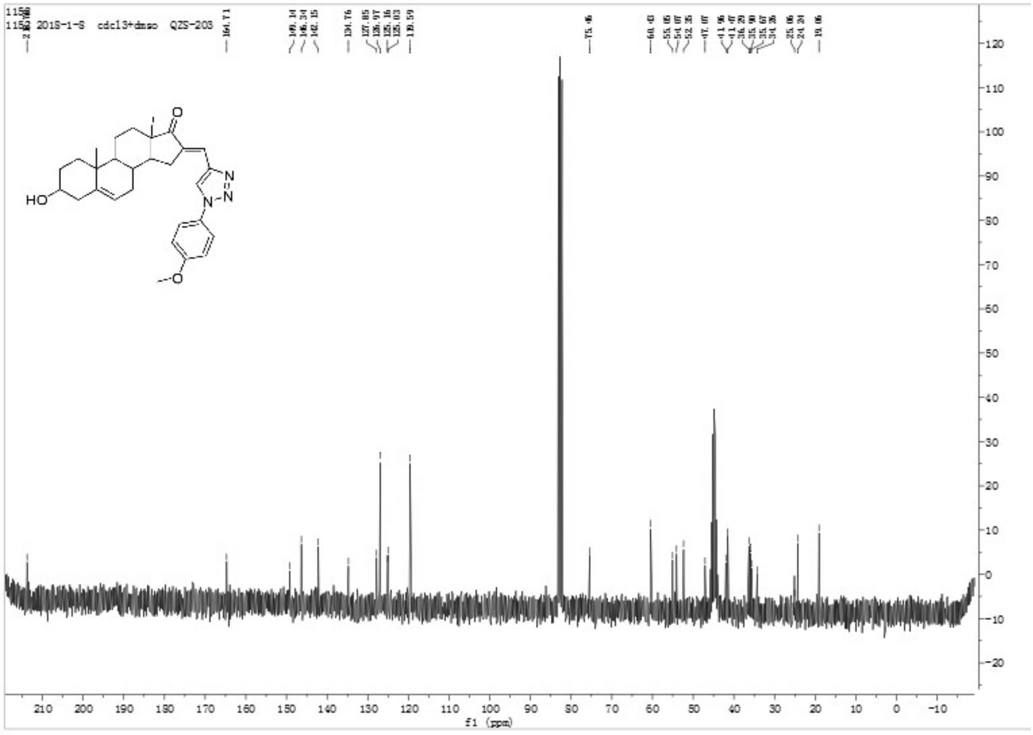

^{1}H-NMR,13C-NMR:(*E*)-3-hydroxy-16-((1-(4-methoxyphenyl)-1*H*-1,2,3-triazol-4-yl)methylene)-10,13-dimethyl-1,3,4,7,8,9,10,11,12,13,15,16-dodecahydro-2*H*-cyclopenta[a]phenanthren-17(14*H*)-one (2s)

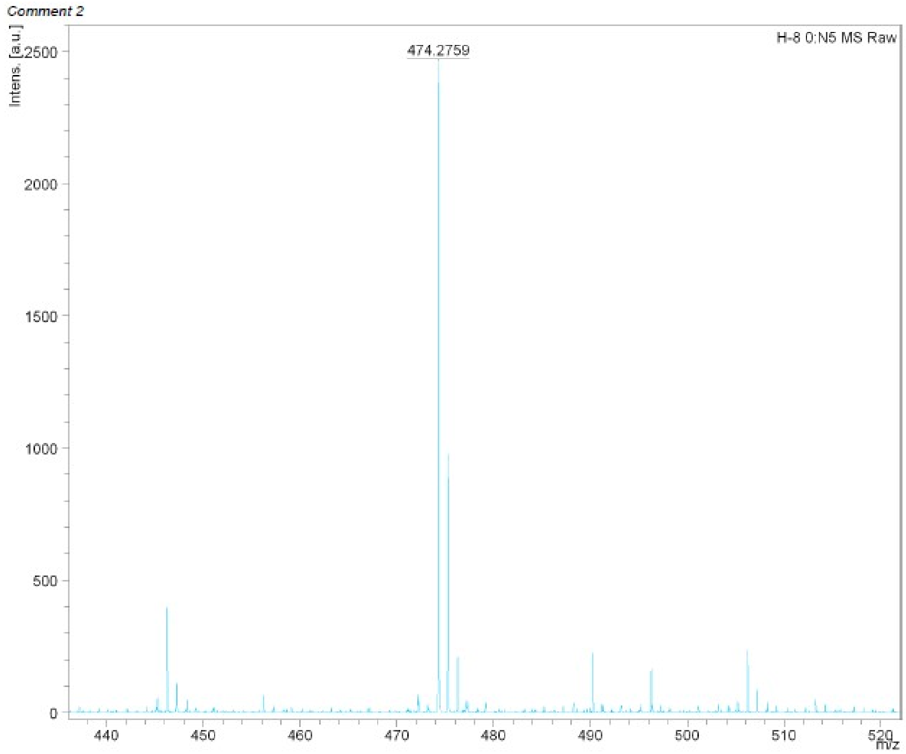

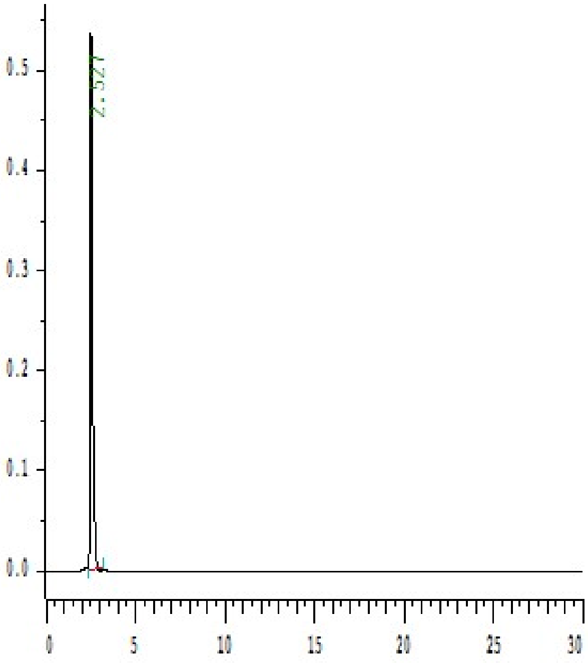

HRMS,HPLC:(*E*)-3-hydroxy-16-((1-(4-methoxyphenyl)-1*H*-1,2,3-triazol-4-yl)methylene)-10,13-dimethyl-1,3,4,7,8,9,10,11,12,13,15,16-dodecahydro-2*H*-cyclopenta[a]phenanthren-17(14*H*)-one (2s)

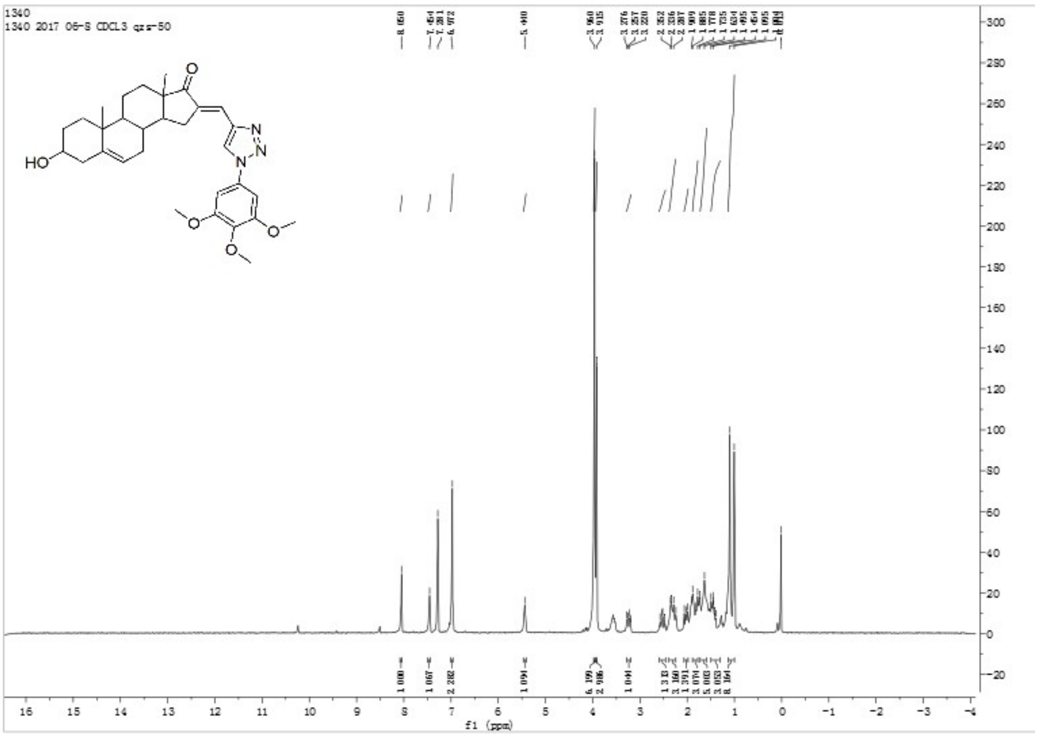

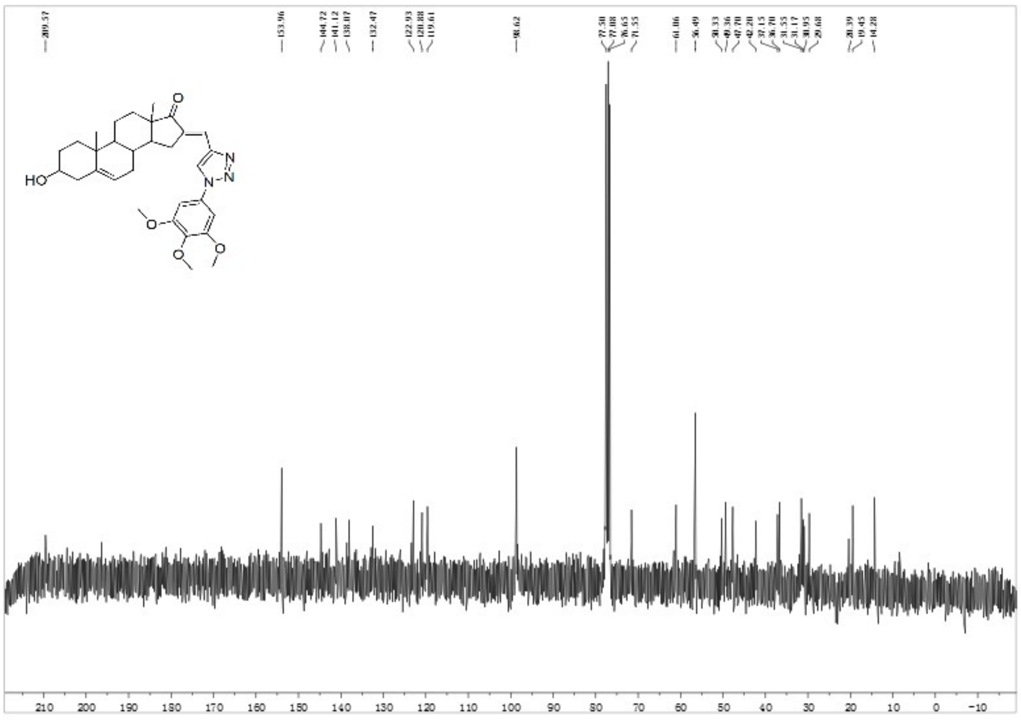

^{1}H-NMR,13C-NMR:(*E*)-3-hydroxy-10,13-dimethyl-16-((1-(3,4,5-trimethoxyphenyl)-1*H*-1,2,3-triazol-4-yl)methylene)-1,3,4,7,8,9,10,11,12,13,15,16-dodecahydro-2*H*-cyclopenta[a]phenanthren-17(14*H*)-one (2u)

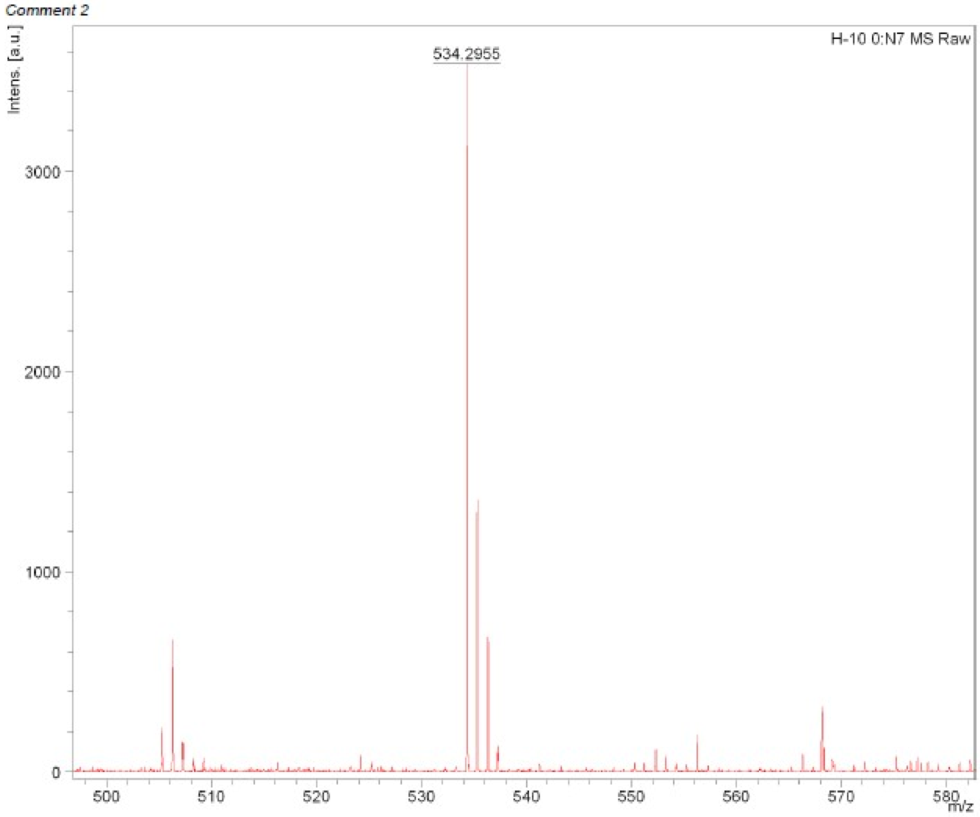

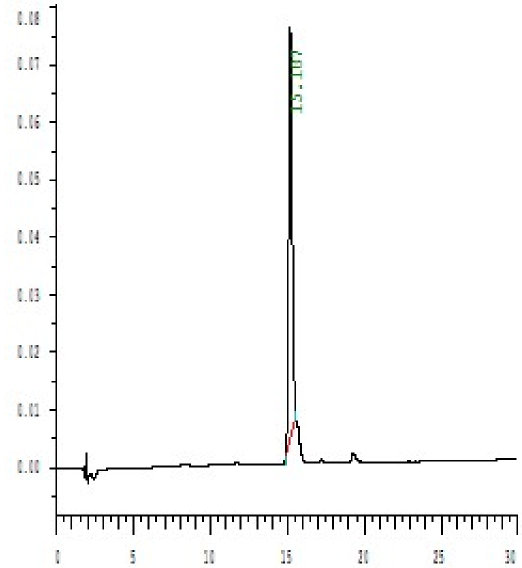

HRMS,HPLC:(*E*)-3-hydroxy-10,13-dimethyl-16-((1-(3,4,5-trimethoxyphenyl)-1*H*-1,2,3-triazol-4-yl)methylene)-1,3,4,7,8,9,10,11,12,13,15,16-dodecahydro-2*H*-cyclopenta[a]phenanthren-17(14*H*)-one (2u)
